# Reproducible Effects of Sex and Acquisition Order on Multiple Global Signal Metrics: Implications for Functional Connectivity Studies of Phenotypic Individual Differences Using fMRI

**DOI:** 10.1002/brb3.70141

**Published:** 2025-04-09

**Authors:** Henry W. Chase, Danella M. Hafeman, Merage Ghane, Alexander Skeba, Tyler Brady, Haris A. Aslam, Richelle Stiffler, Lisa Bonar, Simona Graur, Genna Bebko, Michele Bertocci, Satish Iyengar, Mary L. Phillips

**Affiliations:** ^1^ Department of Psychiatry University of Pittsburgh School of Medicine Pittsburgh Pennsylvania USA; ^2^ Department of Statistics University of Pittsburgh Pittsburgh Pennsylvania USA

**Keywords:** functional connectivity, functional MRI (fMRI), mood disorders, partial least squares, research domain criteria

## Abstract

**Purpose:**

The identification of relationships between individual differences in functional connectivity (FC) and behavior has been the focus of considerable investigation. Although emerging evidence has identified relationships between FC and cognitive performance, relationships between FC and measures of affect, including depressed mood, anhedonia, and anxiety, and decision‐making style, including impulsivity and sensation seeking, appear to be more inconsistent across the literature. This may be due to low power, methodological differences across studies, including the use of global signal correction (GSR), or uncontrolled characteristics of the population.

**Methods:**

Here, we evaluated measures of FC, regional variance, and global signal (GS) across six functional MRI (fMRI) sequences of different tasks and resting states and their relationship with individual differences in self‐reported measures of symptoms of depression, anxiety, impulsivity, reward sensitivity, and sensation seeking, as well as demographic variables and acquisition order, within groups of distressed and healthy young adults (18–25 years old).

**Findings:**

Adopting a training/testing sample structure to the analysis, we found no evidence of reproducible brain/behavior relationships despite identifying regions and connections that reflect reliable between‐scan individual differences. However, summary measures of the GS were reproducibly associated with sex: The most consistent finding was an increase in low frequency variance of the blood‐oxygenation‐level‐dependent (BOLD) signal from all gray matter regions in males relative to females. Post hoc analysis of GS topography yielded sex differences in a number of regions, including cerebellum and putamen. In addition, effects of paradigm acquisition order were observed on GS measures, including an increase in BOLD signal variance across time. In an exploratory analysis, a specific relationship between sex and relative high‐frequency within‐scanner motion was observed.

**Conclusions:**

Together, the findings suggest that FC relationships with affective measures may be inconsistent or modest, but that global phenomena related to state and individual differences can be robust and must be evaluated, particularly in studies of psychiatric disorders such as mood disorders or ADHD, which show sex differences.

## Graphical abstract

We examined multivariate relationships between questionnaire and demographic variables with functional connectivity and regional variance across two samples, including distressed participants. Although no reproducible relationships between neural and behavioral measures were observed, effects of sex (upper panel) and acquisition order (lower panel) were observed on global signal measures.

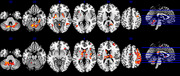

## 1 Introduction

The relationship of symptoms of psychiatric illness with individual differences in the functional connectivity (FC) of neural systems has received widespread empirical evaluation (Rubinov and Bullmore 2013). In the context of disorders, such as major depressive disorder (MDD), bipolar disorder, and anxiety, initial work sought to relate the functional interactions of specific connections between regions to group differences or clinically relevant symptoms. For example, relationships of rumination with default mode network (DMN) FC (Berman et al. 2011), relationships of anhedonia with striatal FC (Felger et al. 2016), and relationships of amygdala FC with anxiety (Jalbrzikowski et al. 2017) have all been described. A general assumption underlying studies of FC is that the (undirected) correlation between regions provides insight into how specific neural regions communicate with one another, that is, transfer information (Sporns 2016). Thus, alterations in FC may represent a disruption of inter‐region or inter‐network information transfer (Fornito, Zalesky, and Breakspear 2015), which may have deleterious consequences for cognition or emotion regulation and thus underlie the symptoms of psychiatric disorders. However, given that there have traditionally been relatively few opportunities to manipulate FC causally (but see Johnen et al. 2015) and observe the effect of induced FC change on behavior, much of the evidence supporting this assumption suffers from limitations associated with correlational evidence, including the potential for confounding. Moreover, more recent evidence emerging from the very large resting functional MRI (fMRI) databases that are now available has challenged findings from the early smaller‐scale studies, perhaps due to a lack of power in such studies (Marek et al. 2022).

More recently, there has been growing interest in relationships between phenotypic measures and network‐level FC summary metrics. Networks are constellations of regions that are consistently connected, structurally or functionally (Sporns 2016). Given that regions in a network show similar functional characteristics, networks are often more plausible units across which to evaluate individual differences than a particular connection (Noble et al. 2022). In fact, recent machine learning studies of FC have supported a relatively dense representation across brain networks, with numerous connections weakly representing information regarding MDD or healthy control groups (Gallo et al. 2023). This would contradict a strong and localized phenotypical representation (i.e., a sparse representation where a small number of connections show large effect sizes), which is implied by seed‐based analyses employing conventional statistical thresholds (Cremers, Wager, and Yarkoni 2017).

It is also possible that some behavioral measures may be more clearly related to individual differences in FC than others. In particular, associations of FC with cognitive performance are commonly observed in substantial samples (Chopra et al. 2022; Cole et al. 2012; Dhamala et al. 2022; Li et al. 2019; Ooi et al. 2022; Reineberg and Banich 2016; Xiao et al. 2023), whereas equivalent associations with affective measures may be smaller and less reliable (Dhamala et al. 2023; Ooi et al. 2022; but see Wan et al. 2020). However, in the context of psychiatric populations, there is a prominent example of the mapping of anhedonic and anxious symptoms onto distributed patterns of FC in substantial samples (training/testing) of patients with MDD (Drysdale et al. 2017) and healthy controls. The study employed canonical correlation analysis (CCA) to evaluate multivariate relationships between brain (FC) and behavior (symptom dimensions of depression obtained from the Hamilton Depression Scale [HAMD]). In the training sample, the authors selected FC measures based on their association with HAMD and then identified two brain/behavior factors reflecting independent mappings of anxiety and anhedonia symptoms onto FC using CCA. These mappings were subsequently replicated in an independent test sample.

As with many other neuroimaging correlates of MDD (Muller et al. 2017), there remain concerns about the reproducibility of these findings. Although differences in methodology and samples can complicate comparisons of different studies, the literature as a whole might be used to focus on a set of plausible descriptions of the most likely underlying phenomena. Specifically, the Drysdale finding was not readily replicated in a new samples using similar techniques (Dinga et al. 2019), whereas large‐scale studies examining resting state FC have suggested very modest effect sizes relating connectivity measures to groups (Anderson et al. 2020; Zhukovsky et al. 2022). Similarly, large‐scale machine learning studies of MDD versus healthy controls suggest modest but above chance (∼60%) decoding accuracy of group (Gallo et al. 2023). Together then, the extant literature supports the view that functional connectomes do carry some reproducible information about depression symptomatology, but that this information may be modest and rather densely represented across the connectome, rather than being related to a well‐defined connection or discrete network. However, the extent to which methodological factors, including assessment of symptom heterogeneity, are responsible for variation in the magnitude of effects across cohorts remains unclear.

Beyond the development of new machine learning tools, there are two significant limitations of the existing literature. First, many of the larger scale studies include clinical measures assessing general symptoms (e.g., HAMD) rather than specific measures of different traits or symptoms with excellent psychometric properties (e.g., Rizvi et al. 2016). Second, the test–retest reliability of individual connections can be variable and is often modest (Noble, Scheinost, and Constable 2019). Unless any fluctuations in FC (e.g., order effects), which might be responsible for low reliability, can be effectively modeled in an analysis, low reliability sets an upper limit on the capacity for replication of individual differences (Vul et al. 2009).

In the present study, we sought to address these limitations in two ways. First, within groups of young adults seeking help for distress and healthy control participants, we collected numerous questionnaires broadly reflecting reward sensitivity and impulsiveness, as well as clinical measures assessing anxiety and depression. This allowed us to examine a detailed multidimensional model of traits/symptoms relevant for mood disorders, including anhedonia (Rizvi et al. 2016), impulsivity (Swann et al. 2008), and behavioral approach/inhibition (Alloy et al. 2008). Our approach to recruitment provided a relatively large range of variation in these measures, which is beneficial for identifying continuous, dimensional relationships. Second, for each participant, we combined FC measures from four different fMRI paradigms, yielding six separate fMRI scans and ∼45 min of multi‐band fMRI data per participant. Each scan underwent considerable nuisance correction, and in the case of task‐based fMRI data, we also corrected for the predicted effects of the task (see Elliott et al. 2019; Fair et al. 2007). We expected to observe that this increase in within‐subjects data would provide more accurate and stable estimates of FC per connection/participant, which would provide a more powerful test.

Using a training/testing strategy (Drysdale et al. 2017) with partial least squares (PLS), we predicted that we would observe that FC and regional variance within a network of regions, including the DMN (e.g., the ventromedial prefrontal cortex) and striatum, would show significant and reproducible relationships with anhedonia and impulsivity, and that amygdala and anterior cingulate cortex connectivity would relate to anxiety and behavioral inhibition. We also paid particular attention to within‐scanner motion and to different metrics representing global signals (GSs). GS regression (GSR) is a key step in many FC analysis pipelines as a way of mitigating motion and physiological noise, but recent evidence suggests that may carry individual differences relevant for psychopathology (see Section 2). It is therefore necessary to evaluate ancillary hypotheses to confirm the assumptions underlying this method. We therefore also evaluated the relationship of GS metrics with motion, paradigm order, behavioral, and demographic variables, as well as the impact of GSR on the findings.

## 2 Methods

### 2.1 Data Collection

#### 2.1.1 Participants

The participant populations for both samples reflected the demographics of Pittsburgh and the surrounding area. The study protocol was approved by the University of Pittsburgh Institutional Review Board. After a complete description of the study to the individuals, written informed consent was obtained. Data from these samples have been published in previous publications (e.g., Bertocci et al. 2023; Chase et al. 2017; Greenberg et al. 2017; Soehner et al. 2019). Demographic information (age, gender, and years of education) and final participant numbers are reported in Tables 1 and 2. All participants were right‐handed and English speaking.

**TABLE 1:** Summary of hypotheses and the statistical tests run to evaluate them.

	Independent variable(s)	Dependent variable(s)	Statistical method	Correction
H1: Demographic/Behavior relationships with GS Measures	Behavior/Demographic variables	Mean correlation/median variance/GS time series per frequency (*n* = 18)	PLS Regression including for demographic variables regression analysis +5× motion	PLS: Training: *p* < 0.05 Testing: *p* < 0.05/number of significant findings in training across all selection methods, given train/test weight correspondence Demographic: Training: *p* < 0.05/54 Testing: *p* < 0.05/number of significant findings in training
*H1: Sensitivity test*: effect of “standard” GSR	As above	As above, using GSR‐corrected measures	As above	As above
*H1: Post hoc*: eigenvalue scaling	Significant behavior/demographic variables from H1	*a*/*b*/*c* from PCA using scaled and unscaled time series data (averaged across frequencies, low frequencies only)	Regression including (demographic) variables +5× motion	*p* < 0.05 to confirm role of *a*, *b*, or *c*
*H1: Post hoc*: topography of standard and weighted GS	Significant findings from H1	Voxelwise association with mean regional gray matter time series	Whole‐brain regression including demographic variables +5× motion	Training, testing, both: all *p* < 0.05 (FWE‐cluster corrected)
H2: Linear order effects on GS	Intercept	Linear change of mean correlation/median variance/GS time series per frequency across time (*n* = 18)	Regression including 5× motion	Training: *p* < 0.05/18 Testing: *p* < 0.05/number of significant findings in training
*H2: Post hoc*: eigenvalue scaling	Intercept	*a*/*b*/*c* from unscaled	Regression including 5× motion	*p* < 0.05 to confirm role of *a*, *b*, or *c*
*H2: Post hoc*: topography of standard and weighted GS	Intercept	Linear change in voxelwise association with mean regional gray matter time series across time	Whole‐brain regression including demographic variables +5× motion	Training, testing, both: all *p* < 0.05 (FWE‐cluster corrected)
*H2: Post hoc*: effect of order, task‐only	Intercept	Linear change in voxelwise association with mean regional gray matter time series across time (task‐only)	Regression including 5× motion	Training: *p* < 0.05/18 Testing: *p* < 0.05/number of significant findings in training
*H2: Post hoc*: effects of demographic/behavioral measures	Significant behavior/demographic variables from H1	Linear change of mean correlation/median variance/GS time series per frequency across time (*n* = 18)	PLS Regression including 5× motion	PLS: Training: *p* < 0.05 Testing: *p* < 0.05/number of significant findings in training across all selection methods, given train/test weight correspondence Training: *p* < 0.05/18 Testing: *p* < 0.05/number of significant findings in training
H3a: Multivariate brain/behavior (FC)	Behavior/Demographic variables	Top 30 FC using 4 selection methods	PLS	Training: *p* < 0.05 Testing: *p* < 0.05/number of significant findings in training across all selection methods, given train/test weight correspondence
*H3a: Sensitivity test*: effect of “standard” GSR	As above	As above, using GSR‐corrected measures	As above	As above
H3b: Multivariate brain/behavior (variance)	Behavior/Demographic variables	Top 30 regional variance using 4 selection methods	PLS	Training: *p* < 0.05 Testing: *p* < 0.05/number of significant findings in training across all selection methods, given train/test weight correspondence
*H3b: Sensitivity test*: effect of “standard” GSR	As above, using GSR‐corrected measures	As above	As above	As above
E1: Demographic/Behavior relationships with motion	12 motion measures	Behavior/Demographic variables	PLS for demographic/behavior matrix *t*‐Tests, correlations for demographic	PLS: Training: *p* < 0.05 Testing: *p* < 0.05/number of significant findings in training across all selection methods, given train/test weight correspondence *t*‐tests: Training, testing, both: all *p* < 0.05 uncorrected

*Note*: For all PLS analyses, even if no significant findings were obtained for training data PLS, PLS was still run on the testing data for exploratory purposes.

Abbreviations: FC, functional connectivity; GSR, global signal correction; PCA, principal component analysis.

**TABLE 2:** Demographic and questionnaire variables.

Measure	Trio HC	Trio DS	Trio comparison	Prisma HC	Prisma DS	Prisma comparison
Sex	41/22	35/12	*χ* ^2^ = 1.11, *p* = 0.29	39/16	50/20	*χ* ^2^ < 1
Age	21.52 (1.71)	21.84 (2.20)	*T* < 1	21.85 (2.024)	21.45 (2.097)	*T*(123) = 1.077, *p* = 0.28
NART IQ	108.99 (6.24)	107.039 (8.025)	*T*(108) = 1.44, *p* = 0.15	106.90 (6.84)	109.63 (7.35)	*T*(123) = −2.13, *p* = 0.036
Education level	5.56 (1.12)	5.19 (1.035)	*T*(108) = 1.74, *p* = 0.084	5.64 (1.22)	5.34 (1.12)	*T*(123) = 1.40, *p* = 0.16
HAMD	0.81 (1.51)	15.40 (6.69)	*T*(49.51) = −14.67, *p* < 0.001	1.45 (2.16)	14.11 (5.84)	*T*(91.57) = −16.75, *p* < 0.001
HAMA	0.65 (1.21)	12.57 (6.69)	*T*(48.24) = −12.071, *p* < 0.001	0.98 (1.24)	11.62 (5.56)	*T*(76.36) = −15.42, *p* < 0.001
MASQ90‐AA	18.51 (2.031)	29.83 (12.20)	*T*(47.91) = −6.30, *p* < 0.001	18.25 (2.15)	27.81 (11.36)	*T*(75.21) = −6.89, *p* < 0.001
MASQ90‐AD	50.29 (9.086)	75.38 (15.80)	*T*(68.41) = −9.75, *p* < 0.001	52.33 (12.91)	73.37 (13.036)	*T*(123) = −9.00, *p* < 0.001
MASQ90‐GD mixed	20.60 (3.43)	43.77 (11.90)	*T*(51.74) = −12.94, *p* < 0.001	22.02 (5.13)	39.83 (10.76)	*T*(103.64) = −12.20, *p* < 0.001
MASQ90‐GD depressive	14.97 (2.99)	36.79 (13.26)	*T*(49.50) = −11.077, *p* < 0.001	15.75 (5.27)	32.73 (10.16)	*T*(108.12) = −12.071, *p* < 0.001
MASQ90‐GD anxious	13.40 (1.98)	26.79 (9.70)	*T*(48.87) = −9.32, *p* < 0.001	14.05 (3.23)	25.00 (8.027)	*T*(95.18) = −10.39, *p* < 0.001
SHAPS	18.83 (5.12)	27.40 (7.24)	*T*(78.63) = −6.94, *p* < 0.001	18.69 (5.018)	27.11 (5.90)	*T*(123) = −8.46, *p* < 0.001
Spielberger state anxiety	28.25 (5.96)	47.79 (11.85)	*T*(63.31) = −10.37, *p* < 0.001	30.53 (9.63)	47.21 (9.49)	*T*(123) = −9.70, *p* < 0.001
Spielberger trait anxiety	30.86 (5.44)	55.79 (11.36)	*T*(61.74) = −13.90, *p* < 0.001	32.04 (8.046)	53.49 (8.71)	*T*(123) = −14.13, *p* < 0.001
SSS boredom susceptibility	2.41 (1.75)	3.23 (1.70)	*T*(108) = −2.47, *p* = 0.015	2.42 (1.73)	2.56 (1.85)	*T* < 1
SSS disinhibition	3.83 (2.34)	4.02 (2.67)	*T* < 1	4.02 (2.47)	4.60 (2.39)	*T*(123) = −1.33, *p* = 0.19
SSS experience seeking	5/73 (1.84)	5.23 (1.99)	*T*(108) = 1.35, *p* = 0.18	5.44 (1.69)	5.67 (1.93)	*T* < 1
SSS thrill and adventure seeking	7.22 (2.55)	5.30 (3.16)	*T*(86.37) = 3.42, *p* < 0.001	7.16 (2.44)	5.47 (2.95)	*T*(123) = 3.43, *p* < 0.001
UPPS‐P negative urgency	22.70 (5.64)	31.72 (7.21)	*T*(84.59) = −7.11, *p* < 0.001	21.69 (5.46)	29.94 (6.70)	*T*(123) = −7.40, *p* < 0.001
UPPS‐P lack of premeditation	20.32 (5.45)	20.79 (5.76)	*T* < 1	19.13 (4.36)	20.90 (6.47)	*T*(120.46) = −1.83, *p* = 0.070
UPPS‐P lack of perseverance	17.87 (3.99)	21.81 (4.48)	*T*(108) = −4.86, *p* < 0.001	17.49 (4.14)	22.60 (5.39)	*T*(122.95) = −5.99, *p* < 0.001
UPPS‐P sensation seeking	35.90 (6.99)	32.49 (8.98)	*T*(84.23) = 2.17, *p* = 0.033	35.87 (6.42)	32.66 (6.66)	*T*(123) = 2.72, *p* = 0.007
UPPS‐P positive urgency	20.81 (6.51)	26.55 (9.97)	*T*(74.30) = −3.44, *p* < 0.001	21.67 (6.29)	26.37 (9.41)	*T*(120.11) = −3.33, *p* = 0.001
BIS‐11 attentional	14.24 (3.30)	17.74 (3.64)	*T*(108) = −5.28, *p* < 0.001	14.16 (3.56)	17.74 (3.77)	*T*(123) = −5.40, *p* < 0.001
BIS‐11 motor	21.29 (3.31)	21.55 (4.23)	*T* < 1	19.62 (3.33)	21.51 (5.00)	*T*(120.00) = −2.54, *p* = 0.013
BIS‐11 non‐planning	21.32 (4.45)	22.64 (4.67)	*T*(108) = −1.51, *p* = 0.13	20.18 (4.60)	23.30 (4.89)	*T*(123) = −3.63, *p* < 0.001
BAS drive	11.41 (1.96)	11.55 (2.75)	*T* < 1	11.53 (2.24)	11.19 (2.73)	*T* < 1
BAS fun seeking	12.14 (2.093)	12.11 (2.78)	*T* < 1	12.05 (1.90)	11.69 (2.22)	*T* < 1
BAS reward responsiveness	17.35 (1.68)	17.17 (2.014)	*T* < 1	17.38 (1.69)	16.79 (2.60)	*T*(119.26) = 1.55, *p* = 0.13
BIS inhibition/punishment sensitivity	19.52 (3.074)	23.66 (3.49)	*T*(108) = −6.59, *p* < 0.001	19.76 (3.65)	23.40 (2.97)	*T*(123) = −6.14, *p* < 0.001

Abbreviations: BAS, behavioral activation system; BIS‐11, Barratt Impulsiveness Scale‐11; HAMA, Hamilton Anxiety Scale; HAMD, Hamilton Depression Scale; SHAPS, Snaith‐Hamilton Pleasure Scale; SSS, Sensation Seeking Scale.

A total of 136 18‐ to 25‐year‐olds who were actively seeking help for psychological distress, showing clear symptoms of clinically assessed distress, and/or a Kessler Distress Scale (K10: Kessler et al. 2002) score > 20, irrespective of diagnosis, in the Pittsburgh area were recruited via community advertisement and student counseling services. A total of 133 healthy 18‐ to 25‐year‐olds were recruited via community advertisement and a participant registry (Pitt + Me: https://pittplusme.org/) in Pittsburgh.

Exclusion criteria included history of head injury, neurological, pervasive developmental disorder, or systemic medical disease (from medical records and report by each potential participant); cognitive impairment (Mini‐Mental State Examination score < 24 and estimated premorbid IQ < 85 (NART); visual disturbance (< 20/40 Snellen visual acuity); current alcohol/substance abuse/dependence (including nicotine) and/or illicit substance use (except cannabis) over the last 3 months, determined by Structured Clinical Interview for DSM‐5 (SCID‐V) (and psychiatric records, if available). For the healthy controls only, this was extended to a lifetime history of alcohol or substance abuse. Lifetime/Present cannabis use (non‐abuse levels) was allowed. Urine tests on the scanning day excluded individuals with current illicit substance use (except cannabis); salivary alcohol tests excluded individuals who are intoxicated on the scanning day. Additional exclusion criteria were MRI screening for claustrophobia, ferromagnetic foreign objects, and positive pregnancy test for female individuals or self‐reporting of pregnancy; taking any psychotropic medication or medication combination for > 2 weeks; and/or being medication‐free for less than 3 months prior to recent (2 weeks) medication. Having a previous history of seeking help for psychological distress, for example, any emotional, behavioral, or substance abuse/dependence problems, irrespective of having received a DSM diagnosis or not, was allowed in distressed individuals, as long as psychotropic medication, if previously prescribed, was no longer being taken, and the individual was free from such medication for a minimum of 3 months (or present medication taken for ≤ 2 weeks). Finally, we excluded individuals who had already performed in the card guessing task in another study.

#### 2.1.2 fMRI Data Acquisition

Functional neuroimaging data were collected across two different scanners at the University of Pittsburgh. The first scanner was a 3.0 Tesla Siemens Trio 2 MRI scanner. Blood‐oxygenation‐level‐dependent (BOLD) images were acquired with a multi‐band gradient echo EPI sequence using a 64‐channel coil (18 × 3 slices/3‐factor multi‐band; 2.3 mm isotropic voxels; TR.TE = 1500/30 ms; field of view = 220 × 220 mm^2^; matrix 96 × 96; flip angle 55°, bandwidth 1860 Hz/Px). Structural 3D axial MPRAGE images were acquired in the same session (TR.TE = 1500/3.19 ms; Flip Angle 8°; FOV = 256 × 256 mm^2^; 1 mm isotropic voxels; 176 continuous slices).

The second of the two was a 3.0 Tesla Siemens Prisma scanner. On this scanner, BOLD images were also acquired using a multi‐band gradient echo EPI sequence using a 64‐channel coil (18 × 3 slices, 3‐factor multi‐band; 2.3 mm isotropic voxels; TR.TE = 1500/30 ms; field of view = 220 × 220 mm^2^; matrix 96 × 96; flip angle 55°, bandwidth 1860 Hz/Px). Structural 3D axial MPRAGE images were acquired in the same session (TR.TE = 1520/3.17 ms; flip angle 8°; FOV = 256 × 256 mm^2^; 1 mm isotropic voxels; 176 continuous slices).

Data from four different paradigms were used for the analysis (see Supporting Information section for details): a card‐guessing task (Chase et al. 2017), emotional face *n*‐back (EFN‐Back) (Bertocci et al. 2023), dynamic faces (Fournier et al. 2014), and resting state. The card guessing and the EFN‐Back tasks both had two runs of data each, making six runs of data per participant. Each card‐guessing task run consisted of 330 images, EFN‐Back 240, dynamic faces 504, and resting 180, making 1824 images in all for each participant.

#### 2.1.3 Questionnaire Measures

Data from the following questionnaires, including subscales, were obtained for each participant: Barratt Impulsiveness Scale‐11 (BIS‐11: motor, attention, non‐planning subscales: Stanford et al. 2009), behavioral activation system (BAS: drive, fun seeking, reward responsiveness subscales), and behavioral inhibition system (BIS: Carver and White 1994), Zuckerman Sensation Seeking Scale (SSS‐V: boredom susceptibility, disinhibition, experience seeking, thrill and adventure seeking: Zuckerman 2007), UPPS‐P (negative/positive urgency, lack of perseverance, lack of premeditation, sensation seeking: Whiteside and Lynam 2001), Snaith–Hamilton Pleasure Scale (SHAPS: Snaith et al. 1995), HAMD Scale (Hamilton 1960), Hamilton Anxiety Scale (HAMA: Hamilton 1959), Spielberger State/Trait Anxiety scales (STAI‐T/S: Spielberger 1983), MASQ anhedonia, anxiety, general depression: anxiety, depression, mixed scales (Watson et al. 1995). Several of these scales were log transformed (see Supporting Information section), and all were *z*‐transformed before analysis.

Age, sex, and education were also included as demographic variables for the PLS and topography analyses. Thus, for the PLS analysis, we had 29 behavioral/demographic variables in all.

### 2.2 Data Preprocessing

Data preprocessing of neuroimaging data employed fMRIPrep—details are reported in the Supporting Information section.

#### 2.2.1 Nuisance Regression

In the first instance, we aimed to perform effective nuisance correction, as far as possible, without GSR. We developed a modification of the CompCor algorithm (Behzadi et al. 2007; Fournier et al. 2014) using a two‐stage process. The first part involved elimination of spikes. First, we made a CompCor mask using a white matter mask and identified high standard deviation voxels (top 2%), as well as ensuring that the voxels did not lie in the amygdala or striatum. Time series from these regions were included in a principal component analysis (PCA). Using the derived component time series for the top 30 components, we identified those with high kurtosis (> 4.5) and any timepoint with an absolute *z* score > 4.5 and isolated the spikes from those time series by only including datapoints from the component time series with (absolute) |*z*|> 3, creating time series consisting only of spikes. These were then combined with spikes identified by a spike‐detection algorithm (Gratton et al. 2020), identifying motion spikes with > 1 mm detrended framewise displacement (FWD) via a high frequency filtering technique. In order to avoid potential collinearity of spikes, we performed PCA on all the CompCor and motion‐derived spike time series and regressed these out of the whole‐brain data. For the five non‐rest fMRI runs, we concurrently regressed out task effects at this stage for each sequence/subject (see Supporting Information section for details).

The second stage of the modified CompCor algorithm involved a standard CompCor analysis using the same mask at the first stage to identify the time series of interest and then performing PCA on these time series. The top seven components were selected, and the derivatives of each were calculated.

For the motion time series, we took the six standard motion parameters (*X*/*Y*/*Z* translation and roll/pitch/yaw rotation) and added a detrended FWD time series to make a matrix of seven time series. These were then filtered to remove very low frequencies (stopband frequency 0.001 Hz), and a matrix was calculated of the basic and square root of the absolute motion time series (*n* = 14), and the first and second derivatives of these were calculated. This matrix of the resulting 42 time series was normalized (*z* score) and submitted to a PCA. The square root of the absolute motion time series was used rather than the squared motion time series as it was better distributed for the purposes of the PCA, with motion spikes having a relatively smaller impact on the PCA solution. The top seven PCs that explained the most variance in the matrix were included in the general linear model (GLM). The goal of this approach was to obtain orthogonal estimates of motion as regressors, given that the raw motion regressors are strongly correlated with one another and therefore may be inefficient in modeling motion‐related spikes. The use of PCA to reduce the number of regressors to seven was to capture the main motion‐related variation while reducing the likelihood of overfitting by including very large numbers of regressors.

These seven motion time series were combined with the 14 CompCor + derivative time series and regressed from the fMRI data for each sequence/subject.

Regional time series were then obtained in the CompCor‐corrected data for each sequence/subject using the Shen parcellation of 278 regions in MNI space (Shen et al. 2013). The resulting time series were then decomposed into six wavelet scales using the maximal overlap discrete wavelet transform (MODWT), using the WMTSA MATLAB toolbox (https://atmos.uw.edu/~wmtsa/ (Percival and Walden 2000). We used the least asymmetric “la8” wavelet approximately corresponding to the following frequencies (Scale 1, 0.33–0.17 Hz; Scale 2, 0.17–0.08 Hz; Scale 3, ∼0.08–0.04 Hz; Scale 4, ∼0.04–0.02 Hz; Scale 5, ∼0.02–0.01 Hz; Scale 6, ∼0.01–0.005 Hz). For the purposes of the FC and variance analyses, we focused on Scales 3–5, although for the GS analyses, we evaluated all six as there is evidence that the GS relates to broadband neural signals (Wen and Liu 2016). For the FC analysis, we calculated the wavelet correlation among each pair of regions for each of the three scales of interest and then *z*‐transformed it using the degrees of freedom provided by the toolbox. Variance measures, representing the variance in the time series at each scale, were obtained for each region and log transformed.

### 2.3 Motion Analysis

In order to include as much data as possible, we did not exclude any participants on the basis of motion (all participants had within‐sequence mean FWD < 1.5). Although it is typical to regress out mean FWD at the between‐subjects level (Yan et al. 2013), during pipeline development, we noticed that mean score provided a somewhat limited view of motion: The FWD time series can be more or less skewed, showing spikes as well as temporal structure (autocorrelation). It is therefore possible that, for example, the canonical distance‐dependent relations of motion and connectivity (e.g., Mahadevan et al. 2021) might be differentially related to different statistical properties of motion (e.g., median vs. peak FWD). Consequently, we generated 12 different metrics of motion from each FWD time series (per sequence): maximum FWD (log transformed); mean FWD; median FWD; FWD standard deviation; FWD skewness; ratio of the mid‐frequency (Scales 3–5) variance to the total variance of FWD; ratio of high (scales 1–2) to low frequency (scale 6) variance of FWD (log transformed); shape parameter “A” and scale parameter “B” (log transformed) of a gamma distribution fitted to FWD. Finally, Matlab (wfbmesti) includes three different ways of computing the Hurst (H) index of fractional Brownian motion (https://www.mathworks.com/help/wavelet/ref/wfbmesti.html), and all three were used here (Types 1–3).

For each metric, we calculated the mean score across all six runs and submitted this to PCA for the Trio and Prisma datasets independently. This approach provided orthogonal components representing different aspects of between‐subjects motion, and we regressed out five components for each FC/variance measure for the Trio/Prisma data independently. Five components were selected due to a preliminary analysis using PLS (see Table 1, E1): We were able to predict significant variation in FC from motion if fewer than five components were regressed out. Five motion regressors were also used as between‐subjects nuisance measures for the GS analyses. We expected that this would reduce the impact of motion‐related factors, which may confound individual differences analysis (Power et al. 2020).

### 2.4 Analysis Strategy

#### 2.4.1 Summary Measures of GSs

GSR is often performed as a form of nuisance regression during the preprocessing of functional connectomes, on the grounds that it is a highly effective way to remove motion‐related and physiological artifacts (Satterthwaite et al. 2019). However, by definition, it is not independent of activity within individual gray matter seeds, which can create statistical problems for the interpretation of connectivity (Murphy and Fox 2017). Moreover, GSs can reflect factors relevant for psychopathology, including arousal/wakefulness (Soehner et al. 2019; Wong et al. 2013), and has been shown to differ across psychiatric groups (Anticevic et al. 2014; Zhang et al. 2019). A further problem is that if phenotypic information is represented in a distributed fashion (Gallo et al. 2023), GSR might have a relatively greater impact than if a signal region is critical.

Given the complexities around GSR, several relatively independent approaches have been developed as alternatives to GSR (Saad et al. 2012) or as ways to evaluate the GS (Anticevic et al. 2014; Wong et al. 2013). Here, we adopted a combination of these methods as well as extending them further to assess the number of global sources. In general, these methods focused largely on gray matter signals, although one included white matter and CSF voxels. Overall, the objective was to evaluate the potential for confounding of individual connections and their hypothesized relationships with behavior by GSs. Toward this end, we calculated several metrics that we expected would assess different aspects of the GS across all six wavelet scales. First, we calculated the mean of the entire distribution of FC measures, that is, across all connections per participant/per wavelet scale across the Shen regions (“mean correlation”). This metric would give an indication of the general tendency of different regions to be positively correlated (Saad et al. 2012). Second, we calculated the variance of the GS time series across all voxels within the whole brain mask per participant/per frequency, then log transformed (‘GS time series variance’). Initially, this was examined in the raw image data, that is, after normalization but prior to nuisance correction. This measure is akin to that employed by Wong et al. (2013), although it uses the wavelet transformation to provide frequency‐specific measures of time series variance. Finally, we calculated the median variance across all of the Shen regions per participant/per frequency, which would give an estimate of the overall amplitude of fluctuations across all gray matter regions (“mean regional variance”). Given that each subregion of a parcellation template is expected to show relatively coherent activation, the amplitude of these fluctuations would avoid suppression of signal caused by averaging across uncorrelated or anti‐correlated regions. Mean correlation and median regional BOLD variance measures were log‐transformed, and all were *z*‐transformed relative to the group before analysis.

This matrix of GS measures was analyzed using the questionnaire and demographic measures via PLS (see Section 2.4.2 for details). All GS measures were corrected for the influence of five PCA‐derived motion parameters before analysis with PLS. In addition, individual investigation of demographic factors, including sex, education, and age, was performed, as these were not expected to relate strongly to the questionnaire factor structure. For the demographic analyses, a regression model was fit, using the independent measure of interest (e.g., sex) as well as five PCA‐derived motion parameters to predict a given GS measure. If a significant finding was identified in the training (Trio) sample, this was confirmed in testing (Prisma) sample. This order was selected because the Trio data were collected before the Prisma data. Our correction strategy for the GS summary measures was to correct across all measures of a given construct in the training sample, so correcting across all 18 measures of GS by 3 demographic measures (sex, age, and education) led to 54 tests and a corrected *p* value of 0.0009. Correction for significant findings observed in the training sample was applied to the testing sample, so if five significant findings were observed in the training sample, a corrected *p* value of 0.01 would be used for the testing sample. Findings reaching uncorrected significance (*p* < 0.05) were also reported for completeness.

Sensitivity analyses were performed by calculating the same measures after GSR in addition to the no‐GSR analysis. Similar correction strategies were applied to other analyses (see Table 1). The first GSR‐corrected method—“standard”—calculated the GS time series from a CompCor‐corrected image, which was then wavelet decomposed for the GS time series variance analysis. This time series was also used to obtain GSR‐corrected time series for the mean correlation and median variance analyses. For the topography analysis, a second GSR‐corrected method—“weighted”—obtained the GS time series from a CompCor‐corrected image, for which each voxel had been *z*‐transformed prior to GS calculation. As above, this was then wavelet decomposed to obtain GS time series variance and was also used to obtain GSR‐corrected mean correlation and median variance.

Order effects on the GS were also examined, following Bijsterbosch et al. (2017), who demonstrate the presence of a broadband signal, particularly in visual and somatosensory regions, which increased in amplitude toward the end of a scan. We evaluated whether, across the acquisition order of sequences, such a signal would become clearer across successive sequences, and thus that GS contributions would become stronger across sequences. We evaluated linear effects of time: weighting the first sequence of a scan session by 2.5, the next by 1.5, and so on for each of the six sequences. Change score analyses did not employ log‐transformed data but were *z*‐transformed for PLS analysis.

We also performed a follow‐up analysis to examine individual differences in the topography of the GS (Anticevic et al. 2014). For this analysis, we focused only on the gray matter time series, calculating a mean time series per participant from each of the template's regions. This time series was then included in a first‐level GLM implemented in SPM12, including a 128‐s high‐pass filter, and fit to CompCor‐corrected and spatially smoothed (6 mm FWHM) images for each sequence. The resulting *t* statistic was *z*‐transformed using the degrees of freedom estimated during the GLM fit. Two methods of generating the GS time series were used for this analysis: the “standard” and “weighted” methods described above. In addition, linear acquisition order effects were also examined in this framework.

For the topography analysis, inference was conducted using cluster correction at pFWE < 0.05, with a cluster forming threshold of *p* < 0.001 uncorrected within SPM. The effect of sex was modeled within a multiple regression model, also including effects of age, years of education, and five motion PC weightings. In addition, for the analysis of the combined training/testing samples, we also modeled scanner as well as scanner by sex interactions in order to check whether the previously identified sex effects differed strongly across cohorts. This approach to modeling scanner differences within a regression model may neglect an effect of signal‐to‐noise (SNR) differences among scanners may have on heteroskedasticity. Although different scanners were used for the training/testing cohort acquisitions, properties of the BOLD acquisition sequences were similar. A simple measure of temporal SNR (GS mean divided by its standard deviation) (Murphy, Bodurka, and Bandettini 2007) was obtained using the raw GS measures extracted after normalization but prior to nuisance correction, averaged across all six sequences, and revealed a trend‐level difference (Trio SNR: mean = 305.42, S.D. = 86.39; Prisma SNR: mean = 327.13, S.D. = 97.17; *t* = 1.80, df = 233, *p* = 0.07). In addition, Breusch–Pagan tests of heteroskedasticity conducted using ROI data suggested that the variance of data from each scanner was largely similar and thus that the regression models employed were appropriate.

We performed a further follow‐up analysis of the GS by performing a PCA of the time series (post‐CompCor correction but not GSR‐corrected) by region matrix for each sequence. We modeled the resulting eigenvalues using a power law function (pow2 in MATLAB), such that the magnitude of an eigenvalue (EVM) was related to its number (#EV) via the following equation:

$$\mathrm{EVM}=a{\left(\#\mathrm{EV}\right)}^{b}+c$$

In general, this model fitted the eigenvalues effectively, with all *R* ^2^’s > 0.88, typically in the high 0.9's. We expected that this analysis would reveal the role of complexity versus scaling in determining the magnitude of the GS. Specifically, if there are numerous orthogonal sources of variation across all regions, that is, there is high complexity, we might expect these to cancel out at the level of the GS, reducing its magnitude. Greater complexity would be reflected in the “*b*” parameter and would be expected to be consistently high whether the time series are *z*‐transformed or not before the PCA. On the other hand, the GS might be larger because the largest eigenvalues show an increased scaling: We would expect this to be reflected in the “*a*” parameter and be removed by *z* transformation of regional time series.

#### 2.4.2 Intra‐class correlations (ICCs) and PLS

Given the number of regions/connections, some selection method was needed to pick FC measures, which would be most likely to carry reliable information pertaining to individual differences in behavior (Drysdale et al. 2017). We generated 4 different selection methods to pick 30 brain measures per analysis. First, for the “correlation” method, we picked the 30 measures (mean FC across sequences) with the largest absolute correlations of brain measures with any of the 26 self‐report measures (demographics not included). Second, for the “ICC” method, we picked 30 mean FC measures with the largest ICC(3,1). Descriptive statistics for the ICC measures for the FC and variance measures, their inter‐relationships, and their topographic location are reported in Section 3.4.

Third, for the “combined” method, we picked 30 mean FC measures with the highest combined ICCs and correlations by *z*‐transforming both measures (relative to other measures across all participants) and adding the *z*‐transformed scores together. Fourth, for the “dynamic” method, we picked the 30 measures with the largest difference in correlations with the 26 self‐report measures across the 6 sequences, and the FC change score between the high and low sequences was used as the measure of interest. These selections were performed in the training sample. The same measures (for each selection method) were then picked for the testing sample. The same four methods were also used for variance measures. Note that, for both FC and variance, any measures with an absolute skewness > 1 were excluded prior to selection to avoid measures that might have disproportionate numbers of outliers.

Thus, a given selection method would provide 30 neural variables per subject, which were submitted into a PLS analysis with the 29 behavioral/demographic variables. Significance was assigned to each component via a bootstrapping analysis. First, PLS was run for the actual data, obtaining 10 components, and a metric of *X*/*Y* correlation was obtained for each component following (Monteiro et al. 2016). Next, a null distribution of these component correlations was generated by permuting the matrices 5000 times, and a *p* value for each component was obtained by comparing the veridical correlation to the permuted distribution. Note that we opted to use PLS rather than CCA (Drysdale et al. 2017), as we found, using simulations, that the *p* values obtained using CCA were poorly calibrated for Type I/Type II error given our sample size.

Given that three of the four selection methods were selected on the basis of correlations, *p* values obtained in the training (Trio) sample were expected to be inflated. Nevertheless, they provided a metric of the expected dimensionality of the solution: It would be possible for all neural measures to be associated with a single behavioral dimension (e.g., anhedonia) versus the existence of several significant brain/behavior components. Replication in the training (Prisma) sample was defined on the basis of two criteria. First, we examined whether there was any significant *p* value, with significance being determined in terms of the dimensionality of the training sample solution. Second, for any components reaching significance in the testing sample, we sought to determine whether their component loadings (saliences) were similar to those found to be significant in the training sample by evaluating whether the saliences for each measure across both the *X* and *Y* matrices were correlated between the training and testing samples (Pearson's *r*) and whether the component explained a similar amount of variance in the *X* and *Y* matrices. An underlying assumption of these analyses is that there is the potential for factor stability across the neural and behavioral data: In other words, that there are similar correlational relationships between the scales (*Y* matrix) or selected neural measures (*X* matrix) between each sample.

This analysis was performed first on the no‐GS FC and variance data. If any significant components were identified (alpha defined as 0.05), we then sought to corroborate the findings using the GSR‐corrected data as a sensitivity analysis. Findings that were eliminated by using GSR may either reflect the effect of the behavioral variable(s) on the GS, or a relationship with an artifact.

For PLS components to be replicable, the underlying factor structure is required to be replicable, that is, that the correlational relationships among measures are preserved. It is conceivable that only one (very strong) relationship between a single neural measure and a single behavioral measure might be reproduced with a poorly reproduced factor structure, but PLS is not suited for this scenario. As a simple proxy of factor consistency, we examined the correspondence of component loadings from a PCA across the training and testing samples using Pearson's correlations. For example, we examined the relationship of loadings of the first component on each FC/variance measure from the training sample with the first component loading from the testing sample, and so on. Offset relationships (i.e., the first training component with the second testing component) were also evaluated. Note that strong joint brain/behavior associations may stabilize components that are more weakly reflected in separate brain or behavior PCA, but that this analysis of PCA correspondence should nevertheless give an indication of the reproducibility of the general correlational structure of the matrices.

2.4.2.1 General Comments. A summary of the evaluated hypotheses and statistical tests is provided in Table 1. For H1/H2, the approach to correcting for the training and testing samples aimed to provide corrected inference in the training sample and also for the subset of previously identified measures for the testing sample. The goal of post hoc analysis was to use uncorrected tests to help interpret previous findings and rule in or out different explanations. For exploratory analysis, the aim was to identify any uncorrected findings. For H3, three of the four PLS tests in the training sample were biased by pre‐selection, so valid inference was restricted to the testing sample.

## 3 Results

### 3.1 Participants

Following exclusions for missing, misformatted, or incomplete data (*n* = 30), or participants with substantial imaging data loss from regions of interest (*n* = 4), we had groups of 110 for the Trio/training sample and 125 for the Prisma/testing sample (see Table 2). All included participants had data in at least 262 regions: A total of 16 regions were not included in some subjects’ whole brain masks, and so connections with these regions were not included in the analysis (see Supporting Information section for details).

### 3.2 Motion

PCA of the 12‐measure motion data confirmed that the different metrics provided complementary information. Were motion to be well described by a single “magnitude” factor with uncorrelated noise, one would expect the first component to explain the majority of the variance. One would also expect lower components to be difficult to replicate across samples.

Details of the five components are reported in Tables 3 and 4. The first component is characterized by a general increase in motion across measures (e.g., high FWD mean); the second component reflects mid‐frequency motion‐related variance; the third reflects consistently high motion (e.g., high FWD median and autocorrelation); the fourth reflects motion spikes; the fifth reflects consistent, high‐frequency motion. The first component explained about 50% of the variance across the motion measures, suggesting that the use of mean FWD as a covariate may not capture the variety of possible motion‐related effects on FC. Notably, the PCA solution was very similar across samples, with loadings correlating *r* > 0.93 for all five components, suggesting that each component reflects a valid and reproducible dimension of motion.

**TABLE 3:** Training sample motion components (C1–C5) derived from principal component analysis (PCA).

	C1	C2	C3	C4	C5	ICCs
Eigenvalues (%)	0.51	0.20	0.15	0.072	0.022	—
Maximum FWD	0.39	−0.071	0.055	0.19	−0.071	0.60
Mean FWD	0.31	−0.23	0.38	−0.18	−0.011	0.76
Median FWD	0.20	−0.29	0.49	−0.27	−0.28	0.78
STD FWD	0.39	−0.12	0.13	0.051	0.075	0.67
FWD Skewness	0.28	0.11	−0.29	0.54	−0.46	0.38
FWD: Mid‐frequency variance/total variance ratio	0.21	0.44	−0.12	−0.41	0.12	0.45
FWD: High‐frequency variance/low‐frequency variance ratio	−0.22	−0.40	0.15	0.42	0.58	0.31
FWD Gamma distribution shape parameter “*A*”	−0.36	0.023	0.19	−0.20	−0.22	0.53
FWD Gamma distribution scale parameter “*B*”	0.39	−0.071	0.034	0.17	0.10	0.59
FWD *H* parameter type 1	−0.064	0.44	0.47	0.31	−0.028	0.23
FWD *H* parameter type 2	−0.039	0.48	0.45	0.21	0.11	0.28
FWD *H* parameter type 3	0.33	0.23	−0.13	−0.16	0.52	0.45

Abbreviations: FWD, framewise displacement; ICCs, Intra‐class correlations.

**TABLE 4:** Testing sample motion components (C1–C5) derived from principal component analysis (PCA).

	C1	C2	C3	C4	C5	ICC
Eigenvalues (%)	0.51	0.21	0.17	0.063	0.021	—
Maximum FWD	0.39	−0.065	0.062	0.16	−0.081	0.58
Mean FWD	0.29	−0.17	0.44	−0.17	0.020	0.71
Median FWD	0.17	−0.19	0.56	−0.30	−0.31	0.80
STD FWD	0.38	−0.091	0.17	0.070	0.13	0.62
FWD Skewness	0.30	0.047	−0.30	0.44	−0.51	0.38
FWD: Mid‐frequency variance/total variance ratio	0.19	0.49	−0.039	−0.36	−0.051	0.45
FWD: High‐frequency variance/low‐frequency variance ratio	−0.20	−0.45	0.16	0.40	0.41	0.31
FWD gamma distribution shape parameter “*A*”	−0.37	0.039	0.19	−0.24	−0.023	0.53
FWD gamma distribution scale parameter “*B*”	0.39	−0.056	0.053	0.15	0.17	0.59
FWD Hurst index type 1	−0.13	0.42	0.39	0.38	−0.025	0.23
FWD Hurst index type 2	−0.11	0.47	0.36	0.36	0.059	0.28
FWD Hurst index type 3	0.31	0.28	−0.13	−0.13	0.64	0.45

Abbreviations: FWD, framewise displacement; ICC, intra‐class correlation.

**FIGURE 1:**
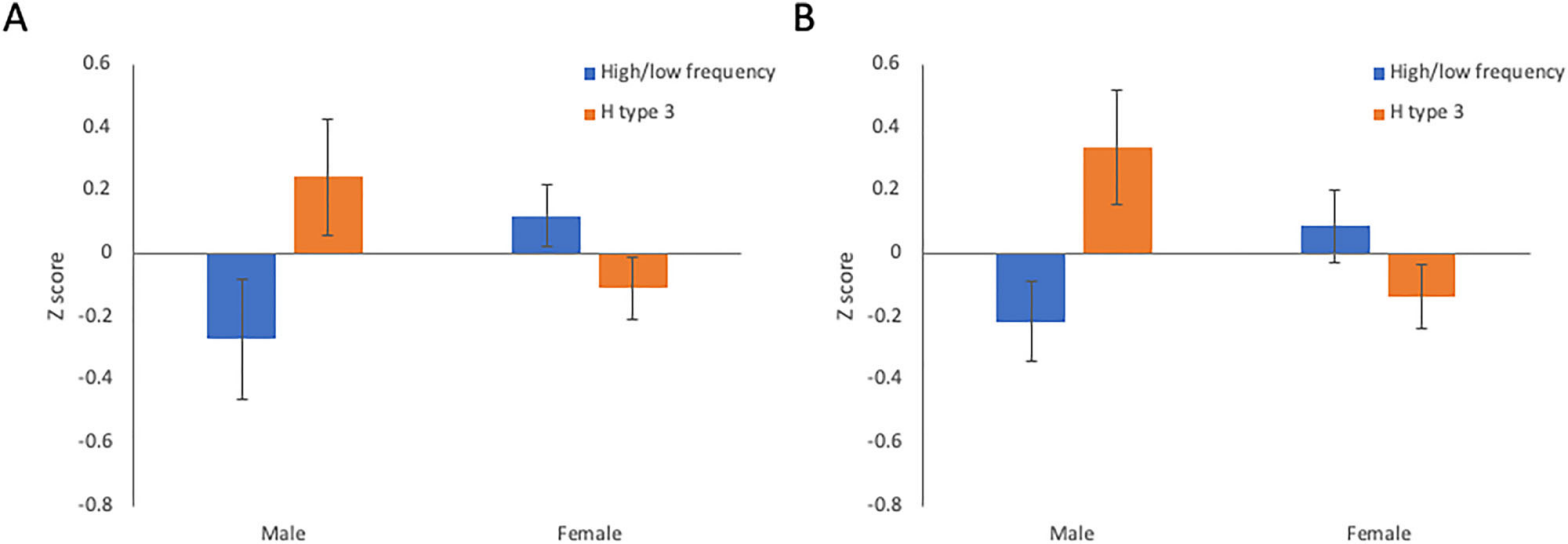
Figures show greater high‐to‐low‐frequency ratio (blue) and lower Hurst index (type 3: orange) in males compared to females in training (A) and testing (B) samples.

A PLS analysis evaluated multivariate relationships between the motion metrics and the 29 questionnaire/demographic variables, but no significant associations were found across either training or testing cohorts (*p*’s > 0.09). In addition, *t*‐test or correlation analyses were performed on each component's loading, evaluating the effect of demographic variables. A significant effect of sex was observed on the fifth component across all participants (both: *t*(233) = 2.84, *p* = 0.005; training: *t* < 1; testing: *t*(123) = 2.55, *p* = 0.012). Another significant finding was observed on the third component (both: *t*(233) = −2.31, *p* = 0.022; training: *t*(108) = −1.28, *p* = 0.20; testing: *t*(123) = −1.47, *p* = 0.14). Post hoc analysis of the scales that contributed to that component showed that relative high‐ versus low‐frequency motion was higher in females than males across both samples, albeit not reaching significance in either subsample (both: *t*(233) = −2.36, *p* = 0.019; training: *t*(108) = −1.92, *p* = 0.057; testing: *t*(123) = −1.54, *p* = 0.13; Figure 1). In addition, Hurst index type 3 was higher in males than females across both samples, although at trend level in the training sample (both: *t*(233) = 2.95, *p* = 0.0035; training: *t*(108) = 1.73, *p* = 0.087; testing: *t*(123) = 2.43, *p* = 0.016; Figure 1). Other measures were not significantly related to sex across all participants (all *p*’s > 0.06). Finally, the two motion variables that were related to sex showed significant inter‐correlation and potentially reflect the same underlying phenomenon: High‐frequency motion showed a negative relationship with *H* parameter type 3 across subjects in both samples (training: *r* = −0.69, *p* < 0.001; testing: *r* = −0.70, *p* < 0.001), even if sex was partialled out (training: *r* = −0.68, *p* < 0.001; testing: *r* = −0.70, *p* < 0.001).

No significant findings relating age and motion variables were identified using correlations, either using the raw variables or the five PCs (*p*’s > 0.06). For education, findings were somewhat inconsistent: Three significant findings (*p*’s < 0.006) were observed in the training sample with the raw motion measures, including skewness (*r* = −0.26, *p* = 0.0060), but these were not replicated in the testing sample (*p*’s > 0.14), with one new finding being observed in the training sample (*p* = 0.045). Using the five PCA measures, scores on Component 2 showed a significant difference in the training sample (*r* = −0.20, *p* = 0.032) but not in the testing sample (*r* = 0.13, *p* = 0.15). When both samples were pooled, the relationship between skewness and education was significant (*r* = −0.19, *p* = 0.0031), with greater education being associated with lower skewness. No significant findings were observed relating education to top 5 component scores generated across both samples, although an association with the third approached significance (*r* = 0.12, *p* = 0.065).

### 3.3 GS Measures

Three primary measures of the GS were obtained per sequence/subject: the mean correlation across all FC measures (per frequency); the median variance across all 278 regions (per frequency); the variance of the GS time series (per frequency).

We performed an initial PLS analysis, relating the behavioral/demographic variables to the primary GS measures. Two significant components, the fourth (*p* = 0.018) and the eighth (*p* = 0.0054), were observed in the training sample. Two significant components were observed in the testing sample (first, *p* = 0.0032 and third, *p* = 0.032), but these did not show a clear similarity to the significant components obtained in the training sample. There were therefore no consistent relationships observed between the primary GS measures and behavioral measures across samples.

As the demographic variables were conceptually distinct from the main dimensions of variation in the questionnaire measures, we evaluated their effects on GS independently. No significant effects of age or education were observed on GS measures (all *p*’s > 0.07).

**FIGURE 2:**
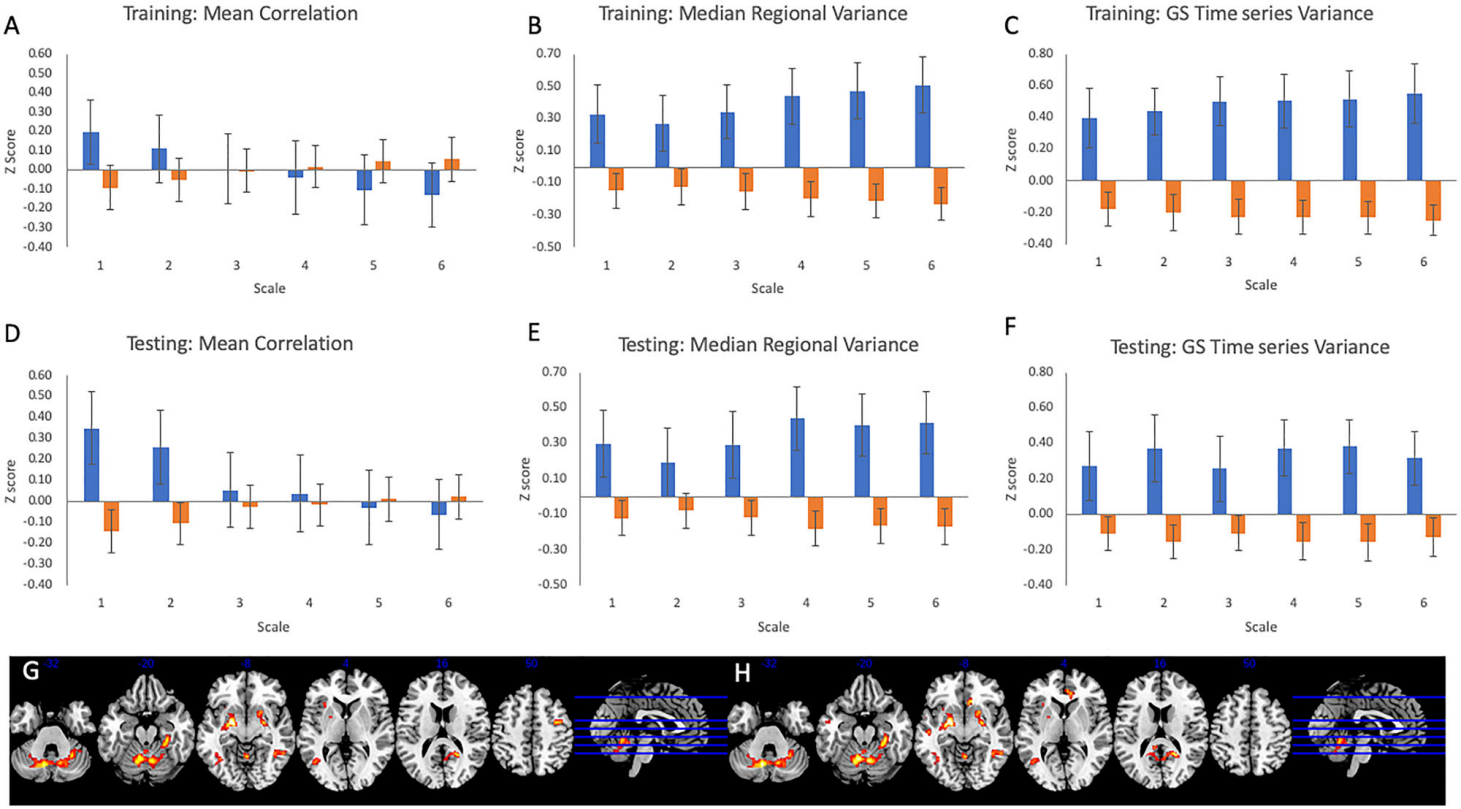
Figures show sex‐related effects of global signal measures (blue = male, orange = female): (A) mean correlation, (B) median regional variance, and (C) global signal time series variance in the training sample; (D) mean correlation, (E) median regional variance, and (F) global signal time series variance in the testing sample; (G and H) topography of sex effects (male > female) on gray matter time series across both samples (G) “standard” method; (H) “weighted” method). Figures are thresholded at *p* < 0.001, *k* = 150 for display purposes.

Multiple regression of sex onto 18 primary GS measures, including five motion components as covariates, yielded seven significant findings at *p* < 0.0009 within the training sample (see Figure 2): two median variances (low frequency bands: *t*’s = 3.58–3.83); and five GS time series variances (*t*’s = 3.59–4.32). Of these seven findings, two replicated in the testing sample at *p* < 0.0071 (*t*’s = 2.86–3.027), with three further at *p* < 0.05 (*t*’s = 2.29‐2.44) and the remaining two non‐significant with *t*’s of 1.38–1.74. Males showed greater median variance and GS time series variance.

In addition, a further 4 of the 18 measures were significant at *p* < 0.05 uncorrected in the training sample: three median variance measures and one GS variance measure (*t*’s = 2.20–3.11). Three of four findings replicated at *p* < 0.05 in the testing sample (*t*’s = 2.070–3.18). One further significant finding was observed in the Prisma sample at *p* < 0.0009, which was non‐significant (mean correlation at scale 1) and one further uncorrected finding at *p* < 0.05 (mean correlation at Scale 2).

In general, the findings in the training sample were similar to those in the testing sample: Across all 18 measures, the effect sizes (Cohen's *d*) obtained in the testing sample, which ranged from *d* = −0.22 to 0.88, were positively correlated (*r* = 0.73) with those from the training sample (ranging from *d* = −0.083 to 0.66) across measures. In summary, median variance and GS time series variance, but not mean correlation, showed consistent associations with sex across both samples.

Sensitivity analysis was conducted with GSR‐corrected mean correlation/median variance and post‐CompCor/motion GS variance. Here, only the two low‐frequency mean variance findings reached corrected significance in the training sample (*p* < 0.0009: *t*’s = 3.71–4.00), both of which replicated at *p* < 0.025 in the testing sample (*t* = 3.27–3.44). A further three findings were observed at uncorrected *p* < 0.05 (*t* = 2.3–3.00), of which two replicated at *p* < 0.05 (*t* = 3.20–3.22). Again, across all 18 measures, the training sample effect sizes correlated with testing sample *t*’s (*r* = 0.83), and no significant GS‐behavior relationships were observed using PLS. Thus, across both tests and samples, low‐frequency median variance was consistently higher in males than females, with effects of sex on GS time series variance being eliminated by CompCor.

Analysis of linear change scores revealed clear evidence of reproducible changes across time: mean correlation decreased across time at all frequency bands (all *p*’s < 0.0028; training *t*’s = −5.044 to −6.79; testing *t*’s = −3.66 to −4.58), whereas the median variance of all ROIs (scales 3–6 all *p*’s < 0.0028: training *t*’s = 3.95–7.62; testing *t*’s = 4.67–7.008) and the variance of the GS time series both increased across time (scales 3–6: training scales: *t*’s = 4.028–7.57), although at trend level in the testing sample (*t*’s = 1.090–2.65, *p*’s > 0.009). No significant relationships between sex and linear change scores of GS‐summary metrics were observed (all uncorrected *p*’s > 0.05 in the training sample and *p*’s > 0.23 in the test sample). Linear change effect sizes were strongly correlated across measures (*r* = 0.92) from training (*d*’s = −0.63 to 0.72) to testing (*d*’s = −0.43 to 0.93).

A PLS analysis of linear change scores with behavior/demographic measures revealed one significant finding in the training sample (3rd component: *p* = 0.0062) and one significant finding in the testing sample (10th component: *p* = 0.040). However, these components did not clearly relate to one another.

Linear order effects using the first five (task) sequences were generally quite similar to those using all sequences, at least as regards median variance and GS time series variance. However, a strong inversion was seen for mean correlation, which increased rather than decreased across all wavelet scales (training *t*’s = 2.38–5.49, all *p*’s < 0.02; testing *t*’s = 4.71–9.35, all *p*’s < 0.001). As previously, no reproducible effects of sex were observed, although some non‐overlapping measures reached uncorrected significance in the training (1 finding at *p* = 0.05) and testing (3 at *p* < 0.02).

#### 3.3.1 GS Eigenvalue Analysis

Two further follow‐up analyses were performed to evaluate these findings in more detail. First, one possible explanation of the GS findings so far is that the BOLD signal is differently scaled in males compared to females, showing fluctuations of a larger amplitude but with no difference in the correlational structure of whole brain correlations (i.e., complexity). This analysis was conducted by modeling the eigenvalues of a whole‐brain PCA (time by region) using a power law function.

Evidence was obtained, which was consistent with the scaling hypothesis: Across all frequencies, the (log transformed) “a” parameter was significantly different between sexes in the training sample (*t*(103) = 2.64; *p* = 0.0093) and trend‐level in the testing sample (*t*(118) = 1.75, *p* = 0.082; see Figure 4). Similar findings were observed in the low frequencies only (training sample: *t*(103) = 3.31; *p* = 0.0012; testing sample *t*(118) = 1.98, *p* = 0.050). By contrast, the “*b*” parameter was not significant in either sample or across all frequencies or low frequencies only (all *t*’s < 1.07). The “*c*” parameter was significantly different in the training sample (all frequencies *t* = ‐2.67, *p* = 0.008; low frequencies only *t* = −4.49, *p* < 0.001), but not in the testing sample (*t* < 1, *t* = −1.53, *p* = 0.13, respectively). Final evidence in favor of the scaling hypothesis was that if regional time series were *z*‐transformed before the PCA, no significant findings were observed across any of the three parameters (all *t*’s < 1).

Consistent with the effect of task order on variance measures seen in the basic GS metrics, the scaling parameter “*a*” obtained from unnormalized data showed a significant linear increase across time (training: *t*(103) = 3.72, *p* < 0.001; testing: *t*(118) = 2.12, *p* = 0.036), whereas the complexity parameter “*b*” did not change significantly (training *t* < 1; testing *t*(118) = 1.88, *p* = 0.062). Notably, the “*c*” parameter reduced across time (training *t*(103) = −7.95, *p* < 0.001; testing *t*(118) = −6.16, *p* < 0.001), consistent with the reduction in the mean correlation seen previously. No significant sex effects were seen on any of these parameters across either sample (all *t*’s < 1).

Together, these analyses suggest that the BOLD signal amplitude is greater in males than females, particularly at low frequencies, but the correlational pattern of findings is largely similar. This analysis suggests that time series from individual voxels/regions might be re‐weighted (e.g., *z*‐transformed) first to avoid different scaling effects across individuals or across the order of tasks in the experimental session.

#### 3.3.2 GS Topography Analysis

In the second analysis, we examined the topography of the global gray matter time series across sexes. Using a “standard” method of creating an unweighted time series by averaging time series across all 278 Shen regions, we evaluated sex differences in GS topography across the whole brain. Findings were somewhat unstable across the training/testing sample, with the training sample showing a trend level difference in the caudate (albeit with a very strong group difference in the peak voxel) and the testing sample showing differences in the putamen and cerebellum. Pooling across both samples, significant findings were observed within the putamen, cerebellum, and left occipital (Figure 2, Table 5). All of these findings revealed a stronger regional representation of the GS in males compared to females—no significant findings in the opposite direction were observed. A non‐significant interaction of sample and sex suggested that sex‐related findings were similar across the training/testing samples.

**TABLE 5:** Coordinates of sex differences in topographic differences of global gray matter time series.

Region	MNI coordinates (*x*/*y*/*z*)	Peak *t*‐statistic	Cluster size (*k*)	Cluster pFWE value
**Training sample: males > females**				
Caudate	6, 8, 14	5.67	103	*p* = 0.14
**Testing sample: males > females**				
Bilateral cerebellum regions V–VIII, Crus I, Vermis VIII	6, −62, −18	4.59	1148	*p* < 0.001
Left putamen	−22, 0, −6	4.76	355	*p* < 0.001
Right parietal	48, −20, 44	4.73	347	*p* = 0.001
Right precentral/inferior frontal gyrus, pars opercularis (IFG)	60, 10, 28	5.15	195	*p* = 0.014
Right putamen	26, −16, −4	3.85	185	*p* = 0.018
**Training & testing samples: males > females**				
Bilateral cerebellum regions V–VIII, Crus I, Vermis VIII, extending into temporal lobe (parahippocampal/fusiform)	28, −38, −22	5.05	2016	*p* < 0.001
Left putamen	−18, −8, −6	5.35	434	*p* < 0.001
Right ventral posterior cingulate	22, −54, 22	5.26	318	*p* = 0.001
Left inferior lateral occipital, middle temporal gyrus	−46, −64, 2	4.11	293	*p* = 0.002
Right temporal/occipital fusiform gyrus, inferior temporal gyrus	42, −52, −6	4.17	216	*p* = 0.011
Right putamen	26, −6, −6	4.82	215	*p* = 0.011
Right precentral gyrus, middle frontal gyrus	36, −6, 48	4.71	152	*p* = 0.046

*Note*: The non‐significant finding in the training sample shown for completeness.

A follow‐up analysis was performed by re‐weighting (*z*‐transforming) each regional time series before averaging during the GS time series calculation: a “weighted” method. This analysis was justified on the basis of the scaling analysis: If some regions show smaller amplitude fluctuations in some individuals but show similar correlational structure across individuals, these regions would contribute less to the GS time series in those individuals. This might contribute to significant topographical differences. However, similar findings were observed, including similar putamen, cerebellum, and occipital regions. In addition, significant differences were observed in the left superior temporal gyrus (*xyz* = −50, −10, −14, *k* = 192, pFWE = 0.016) and the subgenual/pregenual cingulate cortex (*xyz* = 8, 24, −8, *k* = 186, pFWE = 0.019; see Figure 2). This suggests that low signal amplitude in the identified regions of the putamen and cerebellum is not responsible for GS topographic differences in females compared to males, and thus that these regions are simply less correlated with the GS in females compared to males.

**FIGURE 3:**
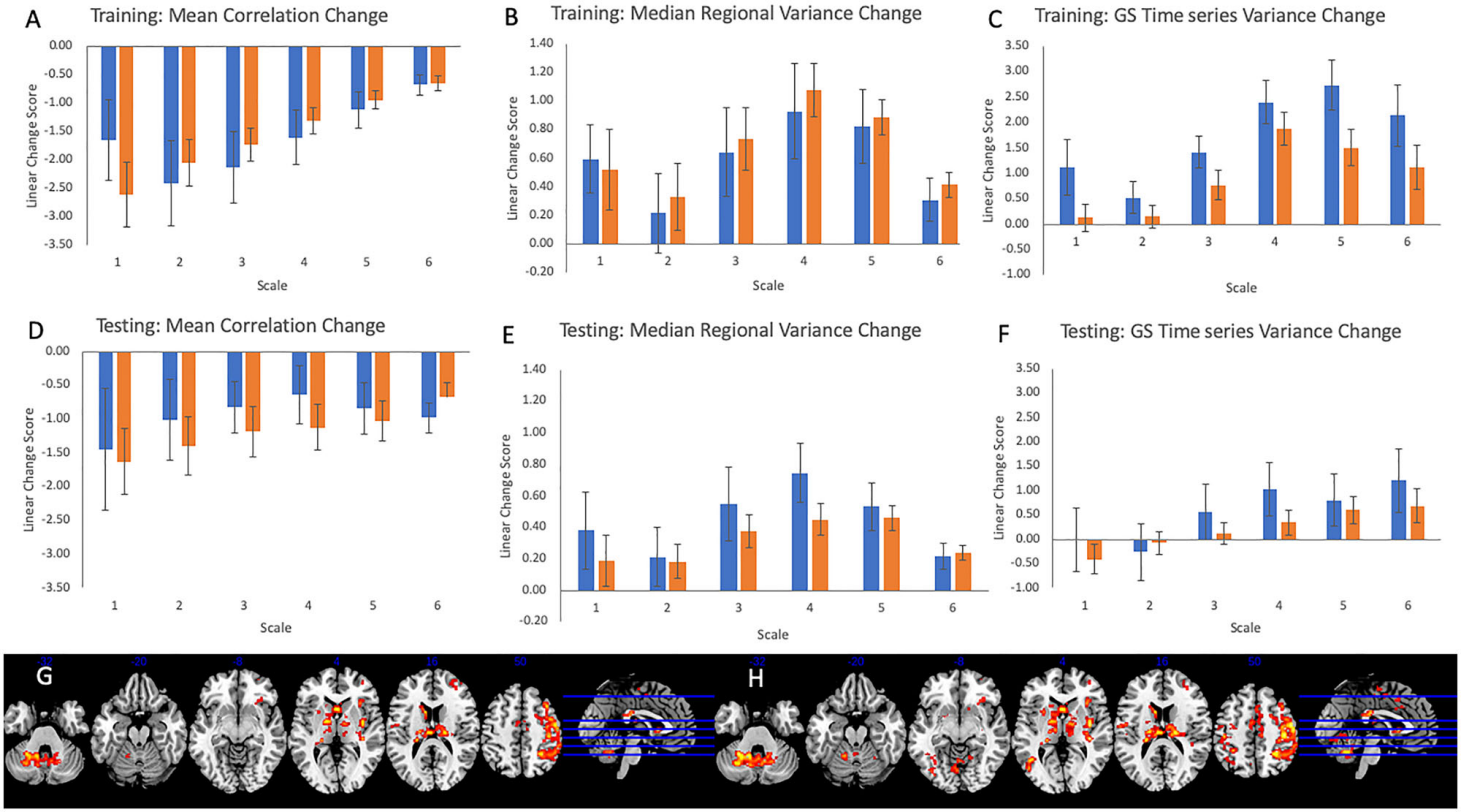
Figures show order‐related effects of global signal measures using linear change scores (positive = increasing, negative = decreasing; blue = male, orange = female): (A) mean correlation, (B) median regional variance, and (C) global signal time series variance in the training sample; (D) mean correlation, (E) median regional variance, and (F) global signal time series variance in the testing sample; (G and H) topography of order effects on gray matter time series across both samples (G) “standard” method; (H) “weighted” method): voxels showing increasing association with the GS time series are displayed. Figures are thresholded at *p* < 0.001, *k* = 150 for display purposes.

A final analysis evaluated the effect of the order of the scan within the session on GS topography. Similar to the previous analysis, significant effects were only seen in the testing sample and both samples, although no significant effects of the scanner were observed. Pooling across both samples, widespread significant linear order effects were observed, such that the GS was more strongly represented in later compared to earlier scans across a number of regions, including a large section of right frontal and parietal cortex, at FWE‐cluster corrected significance, both using a standard and weighted GS (see Figure 3). Further findings were observed in the insula, cerebellum, thalamus, and striatum. No significant regions were observed in which the GS was more strongly represented in earlier compared to later trials. Although significant order effects were observed in regions that had shown sex differences in GS topography in previous analyses (e.g., putamen), no significant effects of sex were observed on the magnitude of linear changes across time in GS topography.

GS topography effects in the first five (task) blocks across both samples were restricted to the right cerebellum, where relationships with the GS became stronger across time (dentate nucleus: standard GS: *xyz* = 20, −60, −36; *t* = 4.59, *k* = 165, *p* = 0.048; weighted *xyz* = 20, −60, −36, *t* = 4.66, *k* = 236, *p* = 0.011). No negative relationships were observed across both samples.

### 3.4 Whole Brain ICCs

The use of six sequences allowed us to compute the reliability of each FC or variance metric using the ICC(3,1) metric. Measures with high ICCs would show a reliable variation of individual differences across the sequences, so an individual showing relatively high FC on one scan would show high FC on another and vice versa.

In the training sample, across all FC measures, the mean ICC was 0.25, with a maximum of 0.77 and minimum of −0.020. The distribution was skewed (skewness = 0.61), with 0.27% of FC measures having an ICC(3,1) > 0.6. A very similar pattern was observed in the testing sample: The correlation among ICCs of all FC measures in the testing sample and ICCs for the same measure in the testing sample was high (*r* = 0.84, *n* = 102,573). Thus, in general, FC measures appear to show a consistent ICC, be it relatively high or low.

ICCs of variance measures were generally much higher than for the FC measures, with a mean of 0.60, a maximum of 0.83 and a minimum of 0.20 for the Trio sample. The distribution was less skewed (−0.31), with 53.47% of regional variance measures having an ICC(3,1) > 0.6. Again, ICCs of variance measures were very similar in the Prisma sample, with a correlation between ICCs for regional variance measures from the Trio scanner with those from the Prisma scanner being high (*r* = 0.84, *n* = 786).

Across both measurement classes (FC/variance), there was some evidence of consistent spatial localization, with different brain regions being associated with relatively high or low ICCs (Figure 5). Notably, regions with more reliable FC, averaged across all connections with that region, were also associated with more reliable variance (Scale 3: training sample *r* = 0.54, testing sample *r* = 0.60; Scale 4: training sample *r* = 0.38, testing sample *r* = 0.53; Scale 5: training sample *r* = 0.34, testing sample *r* = 0.49; all *p*’s < 0.001). Regional reliabilities across scales were highly consistent for FC and variance: regional reliability of FC at Scale 3 was strongly associated with reliability at Scale 4, for example (all *r*’s > 0.79 for FC and variance).

**FIGURE 4:**
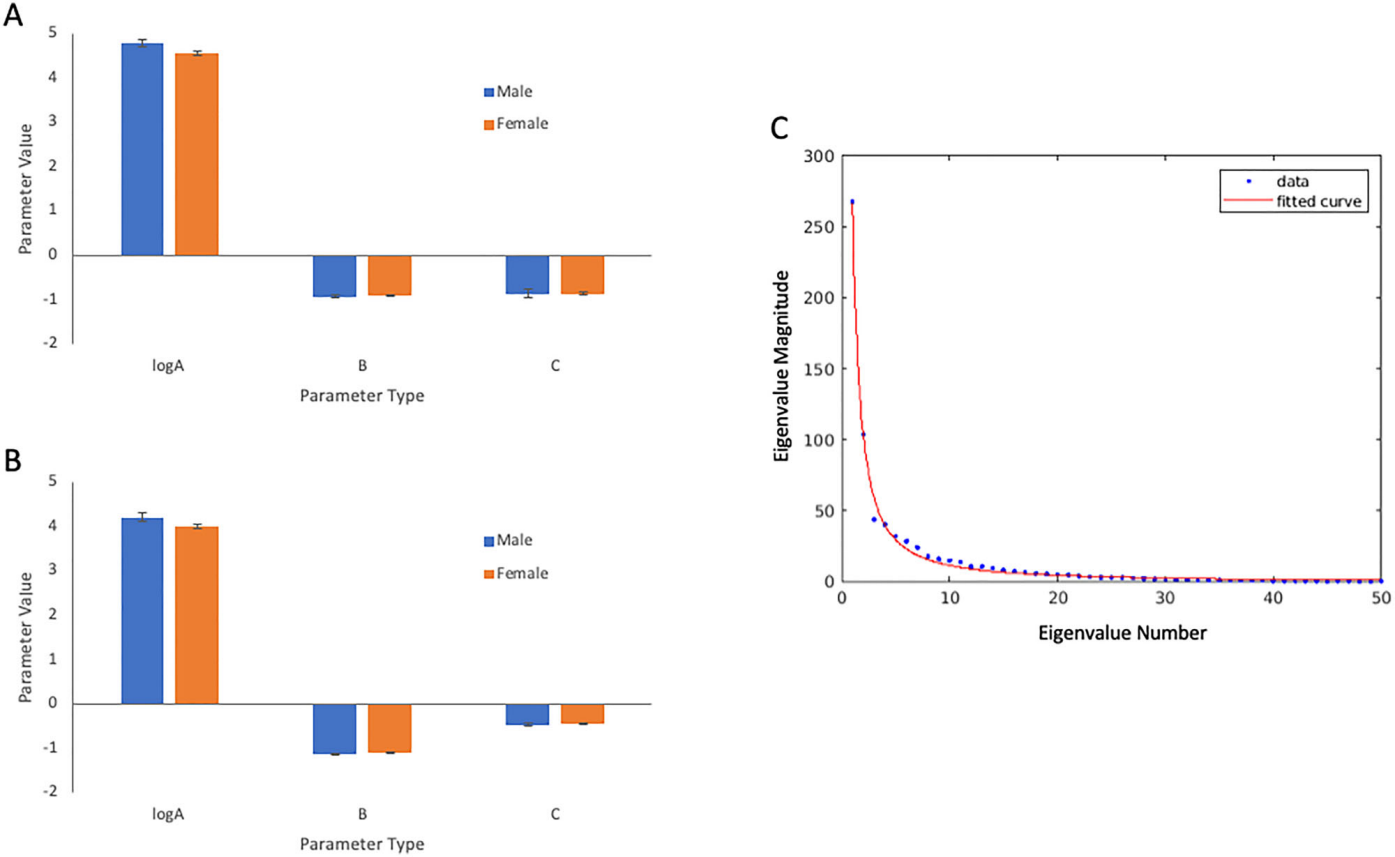
Plots of parameters from the unscaled (non‐*z*‐transformed) eigenvalue analysis: (A) plots of the three power law parameters (log‐transformed a–c) across males (blue) and females (orange) in the training sample; (B) plots of the three power law parameters (log‐transformed a–c) across males (blue) and females (orange) in the testing sample; (C) example of the power law model fit for a given participant/frequency band across a restricted range (*n* = 50) of eigenvalues (for display purposes).

**FIGURE 5:**
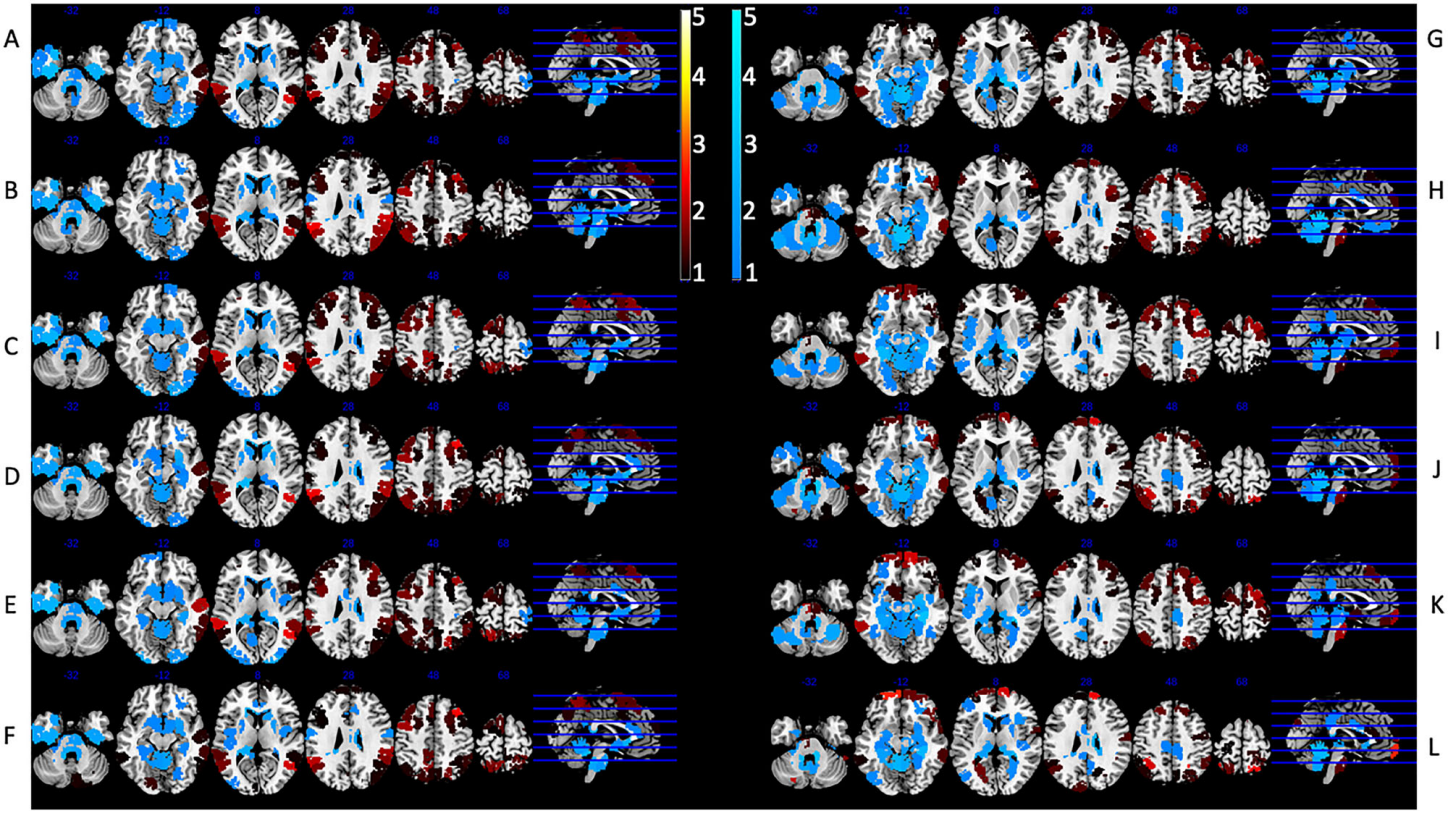
Plots of relative (*z*‐transformed) intra‐class correlations (ICCs) for functional connectivity (FC) and variance measures. Red/Yellow regions represent regions with relatively high (*z* > 1) ICCs, and blue regions represent regions with relatively low (*z* > 1) ICCs: Scale 3 FC ICCs for the training (A) and testing (B) cohorts; Scale 4 FC ICCs for the training (C) and testing (D) cohorts; Scale 5 FC ICCs for the training (E) and testing (F) cohorts; Scale 3 variance ICCs for the training (G) and testing (H) cohorts; Scale 4 variance ICCs for the training (I) and testing (J) cohorts. Scale 5 variance ICCs for the training (K) and testing (L) cohorts.

ICCs of GS measures are reported in Tables 6 and 7.

**TABLE 6:** Intra‐class correlations (ICCs) of global signal (GS) measures, training sample.

	Scale 1	Scale 2	Scale 3	Scale 4	Scale 5	Scale 6
Mean correlation	0.39	0.47	0.46	0.41	0.41	0.32
Median regional variance	0.65	0.66	0.63	0.47	0.45	0.36
GS time series variance	0.62	0.74	0.61	0.53	0.52	0.46

**TABLE 7:** Intra‐class correlations (ICCs) of global signal (GS) measures, testing sample.

	Scale 1	Scale 2	Scale 3	Scale 4	Scale 5	Scale 6
Mean correlation	0.40	0.47	0.40	0.37	0.28	0.25
Median regional variance	0.34	0.52	0.63	0.59	0.50	0.42
GS time series variance	0.62	0.64	0.62	0.63	0.62	0.53

### 3.5 PLS Analysis of FC and Variance

Four selection methods were used to define the neural data to be submitted to the PLS from the Trio sample. Unsurprisingly, PLS revealed that significant brain/behavior components were obtained for the three methods for which selection was based on correlation with behavioral measures (correlation: first 4 components, *p*’s < 0.0015; ICC/correlation: Components 1, 2, and 5, *p*’s < 0.038; delta correlation: all 10 components aside from 6 and 8, *p* < 0.029). However, for the method where neural measures were selected on the basis of ICCs, no significant components were identified using PLS (*p*’s > 0.38). In a post hoc analysis, we observed that, across all FC measures, at all frequencies, the association of an FC measure's ICCs with its maximum absolute correlation with the behavioral measures was very modest (training: *r* = −0.023; testing: *r* = 0.022). Thus, FC measures that carry reliable information about individual differences across sequences are not the same as those that are associated with behavioral measures. Similar findings were observed for the variance measures (training: *r* = 0.14; testing: *r* = −0.019).

Next, we tested whether significant brain/behavior associations could be identified in the testing sample. Given that 15 significant (*p* < 0.05) components had been identified in the training sample, replication of a similar component (i.e., with strong similarity of saliences between training and testing) would require a corrected *p* value of 0.0033. No components were observed at corrected levels of significance. An uncorrected finding was observed for delta correlation (Component 3, *p* = 0.0084), but this component did not correspond clearly to any component in the training sample, showing low or inverted correlations with equivalent neural saliences. All other components from other selection methods did not reach uncorrected significance (correlation *p*’s > 0.16; ICC/correlation *p*’s > 0.053; ICC alone *p*’s > 0.17).

The same analysis was conducted using the variance measures. Findings were very similar: Significant components were identified in two selection methods (correlation: fifth component *p* = 0.038; delta correlation: first, second, and fifth components, *p* < 0.001), but not on the ICC‐based methods (ICC only: *p*’s > 0.10; ICC/correlation: *p*’s > 0.12). Given four components were identified, a corrected significance of *p* = 0.0125 was applied. For the delta correlation, one significant component was observed (fourth, *p* = 0.011) in the testing sample but which was not clearly related to any of the significant training components. No other significant components were observed in the testing sample (*p*’s for all components in the testing sample *p* > 0.07).

Finally, a further sensitivity analysis was performed using (standard) GSR‐corrected measures in order to confirm that GSR did not radically affect the pattern of brain/behavior associations and contradict our findings from the no‐GSR analysis. In general, a very similar picture to the first was obtained, which generally provided a very similar picture to the non‐GSR corrected measures, with largely non‐significant or uncorrected findings in the testing sample. A possible exception was obtained with the correlation‐based selection, in which the first component in the testing sample had a *p* value of 0.008. However, this was higher than the required *p* value to reach corrected significance and did not show a clear correspondence with the relevant neural PLS saliences from the training sample.

For FC, the consistency of component loading was good across training/testing samples for ICC‐selected and ICC/correlation‐selected FC measures, at least for the first three components (ICC: *r*’s > 0.85; ICC/correlation: *r*’s > 0.76). For lower components, a more variable pattern was observed, with some negative relationships but some positive (*r* ∼ 0.5). For the correlation‐selected or delta correlation–selected matrices, there was no consistent correspondence across any of the components: Two substantial anticorrelations were observed (absolute *r*’s > 0.54), but mostly associations were modest (absolute *r*’s = 0–0.4). Similar findings were observed if components were offset by one (e.g., examination the first from the training sample with the second from the testing sample): An occasional large association was observed, but generally associations were modest.

A somewhat different pattern was observed for the variance measures. As with FC, the ICC‐selected matrix showed excellent correspondence, at least for the second and third components (*r*’s > 0.74), with a moderate correspondence for the first (*r* = 0.47), before dropping off sharply. The correlation‐selected and ICC/correlation‐selected matrices generally showed modest associations, with two substantial correlations being observed (absolute *r* > 0.5). The delta correlation–selected matrix showed high associations for the first two components (*r*’s > 0.82) with a more mixed pattern for the remaining components. Examining offset components revealed more large mappings than for FC (e.g., absolute *r*’s = 0.4–0.80), but again generally a mixed pattern. Notably, the first eigenvalue for the variance measures was generally higher for variance (4.45–12.17) than for the FC measures (2.75–6.15), but similar for other components. Overall, the pattern of the variance measures suggested more poorly spatially localized early components with more complex region‐to‐component correspondence than for FC, but with early components with a greater capacity to explain overall variation of regional measures.

Component correspondence was more favorable for the behavior/demographic data across samples, with good correspondence for the first four components (*r*’s = 0.65–0.96) and some correspondence thereafter (all absolute *r*’s > 0.35 for Components 5–10 aside from component 8 *r* = −0.011). Offset component relationships also revealed some substantial associations (absolute *r*’s = 0.52–0.71).

Together, these analyses demonstrate the identification of reproducible brain/behavior relationships would have been possible using PLS, at least for high components in the ICC‐ or ICC/correlation‐selected FC matrices, had substantial associations between brain and behavior components been present. It may also have been possible to demonstrate effects for lower (>3) components, but this may have been hindered by variable component correspondence.

## 4 Discussion

In the present study, we performed a multivariate analysis of brain/behavior relationships using cross‐paradigm estimates of FC in separate training/testing samples. We also performed extensive analysis of the GS. Although we were unable to find reproducible brain–behavior relationships between FC measures and questionnaire measures of depression, anxiety, impulsivity, and reward sensitivity using PLS analysis, we found a reproducible relationship between sex and a GS measure, mean global variance of the BOLD signal at low frequencies, which was very similar across samples and survived sensitivity tests. Two further post hoc tests suggested: First, that this was a scaling phenomenon, with males showing a greater amplitude of fluctuations, particularly at low frequencies. The second post hoc test showed that the GS showed an altered topography across sexes, with females showing a reduced contribution of (gray matter) GS in the putamen and cerebellum. Intriguingly, a recent study by Lotter et al. (2024) has demonstrated the relevance of putamen for the postpartum changes in FC, perhaps suggesting that putamen FC may relate to hormone levels. Notably, these putamen/cerebellum differences were similar whether time series were scaled or unscaled. Although substantial changes were observed, showing changes in the topographic contribution of the GS across time within the scanner, these appeared to be unrelated to sex. Overall, our findings clearly argue against the notion that the GS does not carry any relevant state‐ or trait‐related information, which may complicate its use as a nuisance regressor.

In addition, we also observed associations of sex with relative high‐frequency within‐scanner motion, with females showing more high‐frequency motion than males. There has been a prior suggestion of this within the literature (Gratton et al. 2020), although in that study the finding was not always observed across cohorts in their study and was less consistent than other reported associations (e.g., age/body mass index). We also used a different measure of high‐frequency motion, including a relative measure of high‐to‐low frequency motion, with a further supporting finding of an alteration in the Hurst exponent. A further difference is the amount of data per participant, which varied markedly across different datasets in the Gratton study. The finding may relate to differences in breathing patterns between sexes, which are reflected motion metrics (Lynch et al. 2020). A key point of our study, which is also suggested by the Gratton work, is that within‐scanner motion is not well explained by a single factor representing general increases or decreases but rather may have several different characteristics, including the proportion of high‐frequency motion, spikes, and autocorrelation. To our knowledge, the unique impact of these different properties on FC has not been widely investigated, but it is possible that each may have distinct effects.

Further findings suggested physiological rather than neural underpinning of sex differences in the GS. By physiological, we mean findings reflecting the hemodynamic properties of the gray matter, as well as artifacts including motion, respiratory, and cardiac influences. These factors can be contrasted with the BOLD signal as a proxy of neural signaling. The indications that there is a physiological basis for the sex differences in GS are as follows. First, several analyses suggested that the whole brain BOLD signal was differently scaled in males compared to females, particularly at low frequencies, showing larger fluctuations but similar global integration (i.e., correlation). This observation is consistent with prior research demonstrating enhanced long‐duration temporal autocorrelations in the BOLD signal of males compared to females (Dhamala et al. 2020) in a large, well‐matched sample. Note that, although we fitted a power law function to global time series eigenvalues, this was largely because it provided an effective fit of these data and should not be taken to imply that we are providing support for power law models of neural organization and function (Sporns 2016). Second, strong findings were observed in the variance of the GS time series, but these were not replicated following CompCor/motion correction. This suggests that there may be sex‐specific components to the raw BOLD signal that nuisance correction is able to remove. Finally, the effects of sex on GS were measure‐specific, with the mean FC across all connections showing no differences.

However, a further finding suggested a neural, rather than physiological, basis for sex differences in the GS was the implication of the putamen and cerebellum in GS topography. These findings were also observed if a weighted GS measure was used, suggesting that BOLD signal scaling was not responsible for these findings. Briefly, for the weighting method, we rescaled the intensity of each voxel before GS calculation via *z* transformation, so time series from high‐amplitude regions would not contribute disproportionately to the GS. We would expect this rescaling to impact between‐subject findings too. However, rather than eliminating the regional findings in the putamen and cerebellum, this method appeared more sensitive, and further, previously non‐significant regions were observed, including the subgenual cingulate cortex. Overall, however, the topography findings were somewhat less reproducible than those from the GS summary analyses, as they were not consistently observed across the training/testing samples. Moreover, significant findings using a whole‐brain cluster‐corrected analysis do not necessarily indicate anatomical specificity.

A statistical justification for examining sex is that its effects were large and consistent enough to operate as a confounder in the analyses. A common strategy of using GSR plus including sex as a covariate will effectively account for sex effects, provided that the effect of sex is linearly decomposable: However, it is possible it may differentially affect certain FC measures over others given that we observed clearer sex differences at lower frequencies. It may also affect the relative precision of measurements: if removing the GS removes a relatively greater proportion of the signal in males, noise may have a greater relative influence in the remaining time series, reducing the precision of the estimate. Nevertheless, there was still a suggestion of the efficacy of GSR (see also Li et al. 2019), insofar as the clearest FC/behavior association (albeit uncorrected) was observed following GSR.

We also observed potential state effects on the GS. We examined linear effects across acquisition order and saw a reduction in the mean correlation but increased BOLD signal variance, as well as widespread changes in GS topography. These changes may be related to changes in alertness across the scan session (e.g., Wong et al. 2013). Similar observations of changing FC with increasing time‐in‐scanner have recently been reported by Korponay, Janes, and Frederick (2024) and were ascribed to a changing physiological artifact. In our findings, the contribution of the GS to several cortical and subcortical regions, including the thalamus and striatum, increased during time in the scanner. These changes were broadly similar across sexes, despite the overall differences in GS metrics. These findings suggest that GSR may have different effects depending on when the scan was conducted within scanning session, which may have implications for the reproducibility of findings across studies. For example, it is notable that several of the regions implicated by this analysis, including primary sensorimotor regions and the thalamus, overlapped with “Biotype 4” identified by Drysdale et al. (2017). Given the magnitude of these effects (see also Bijsterbosch et al. 2017), it seems likely that information about the position of a given scan relative to others will need to be accounted for in analysis pipelines. In general, the different GS measures often showed moderate‐to‐good ICCs, suggesting that although these order effects were large, they may be relatively consistent across individuals. One complication in the interpretation of these findings was that the resting scan always came last, whereas the task scans were counterbalanced in order in the first five. Regional variance increased across the task sequences in a similar way to rest, as did the relationship of the cerebellum with the GS time series. However, the widespread changes in relationships between the GS time series and other neural regions were not observed across the task‐only sequences. In addition, different changes in mean correlation were observed in the task‐only sequences versus all sequences. Overall, the position of a given scan within the task sequence does seem to affect GS properties, although the extent to which this is affected by task content cannot be determined in this design. Further discussion of the findings is included in the Supporting Information section.

### 4.1 Limitations

A key limitation of the present study is that the sample size is smaller than that employed by Drysdale et al. Methods such as PLS or CCA can require substantial sample sizes to provide accurate and reproducible estimates of brain/behavior mappings (Helmer et al. 2024). We had expected that the increased specificity of our behavioral metrics and the greatly increased amount of data per participant would mitigate this limitation. If we can assume that this is correct, for example, that SHAPS‐measured anhedonia is more specific than HAMD‐measured depression, then possible reasons for our inability to obtain corroboratory evidence in our sample may be (A) that the underlying effect is small and spatially distributed, and larger samples would be needed; (B) that brain/behavior relationships reported by Drysdale et al. can only be seen in depressed patients with more severe symptoms; and/or (C) that findings are restricted to the resting state and not FC more generally, and task‐based data may be a more effective predictor if modeled using conventional GLMs. Although order was counterbalanced for the different tasks, there were still some fixed features to each sequence, and the resting state was always last.

A further possible limitation of specific measures of symptom and trait measures is that the factor structure of behavior would be unstable as the dimensionality of possible symptom combinations would be very high. This did not appear to be the case, although PLS would tend to identify preferentially components that explained variance across a number of different measures—potentially mitigating the advantages of specific measures. Nevertheless, more specific components might appear in lower ranked components. Methods to enforce sparse solutions may represent a productive alternative approach (Monteiro et al. 2016). Although our behavioral measures covered a wide range of different symptoms, we did not focus on rumination scales, which may be relevant for mind wandering during resting state FC (Berman et al. 2011), or examine other clinical variables such as family history (Chase et al. 2021). The UPPS‐P has some overlap with other questionnaires, which might affect the PLS factor structure (Whiteside and Lynam 2001). Our measures of sex were restricted to simple self‐reports, and a more detailed investigation of birth sex versus gender, as well as biological markers and evaluation across the lifespan, would be needed to evaluate the generalizability of the findings and interpret them in more detail (DeCasien et al. 2022; McCarthy et al. 2012). Finally, data were obtained across different MRI scanners (Trio/Prisma) across the duration of the study. Our analysis approach was built on the assumption that a finding of clinical importance should be demonstrable across different scanners. Although some measures were highly reproducible, the change of scanners may have obscured others. In general, our findings do not rule out the presence of brain/behavior relationships with affective measures but suggest they are not robust to methodological differences in the sample sizes employed here.

### 4.2 Summary

Contrary to our expectations, no reproducible brain/behavior relationships relating individual differences in measures of depression, anxiety, and impulsivity to FC or regional variance measures were observed. However, substantial and reproducible effects of sex and acquisition order on measures of GS were observed. Effects of sex on measures of high‐frequency motion were also observed. These findings have implications for the use of GS and motion regression in the analysis of individual differences in FC, as well as informing analytic approaches to uncovering the neural correlates of the symptoms and traits associated with affective disorders.

## Author Contributions

**Henry W. Chase**: conceptualization, writing—original draft, data curation, formal analysis, visualization, methodology, investigation, writing—review and editing, validation, software. **Danella M. Hafeman**: conceptualization, writing—review and editing, methodology, formal analysis. **Merage Ghane**: writing—review and editing, conceptualization, methodology. **Alexander Skeba**: data curation, formal analysis, methodology, software. **Tyler Brady**: data curation, formal analysis, methodology, software. **Haris A. Aslam**: data curation, project administration, writing—review and editing. **Richelle Stiffler**: data curation, project administration, writing—review and editing. **Lisa Bonar**: data curation, project administration, resources. **Simona Graur**: data curation, project administration. **Genna Bebko**: data curation, project administration. **Michele Bertocci**: data curation, writing—review and editing. **Satish Iyengar**: writing—review and editing, formal analysis. **Mary L. Phillips**: conceptualization, supervision, funding acquisition, resources.

## Conflicts of Interest

The authors declare no conflicts of interest.

### Peer Review

The peer review history for this article is available at https://publons.com/publon/10.1002/brb3.70141.

## Supporting information



Supporting Information

## Data Availability

Data are uploaded to, and will be available through, the National Institute of Mental Health Data Archive (NDA).
