# Supplementary material for: Reproducible Effects of Sex and Acquisition Order on Multiple Global Signal Metrics: Implications for Functional Connectivity Studies of Phenotypic Individual Differences Using fMRI
Source: Brain Behav. 2025 Apr 9;15(4):e70141. doi: 10.1002/brb3.70141 (PMC11979359; doi:10.1002/brb3.70141)
Supplement: Supplementary file 1 — Supporting Information [file BRB3-15-e70141-s001.docx]

Supplement

*Log-transformed questionnaires*

The following questionnaires were log transformed before analysis: HAMA, HAMD, MASQ-AA, the three general distress scales of the MASQ-GD, BIS-11 Motor, UPPS-P lack of premeditation, positive urgency.

*Missing ROIs*

Sixteen of the 278 regions were excluded from the PLS analysis due to insufficient data in some participants. Thirteen of those regions were in the cerebellum, primarily in ventral regions. A further three were in the posterior occipital lobe (primary visual cortex: x1), ventral temporal lobe (x1) and the brainstem (x1). Note that data from these regions were included in the GS and eigenvalue modelling analyses if available for a given participant.

*Task descriptions*

**Card Guessing Task:** During each trial of the card guessing paradigm (Chase et al., 2017), individuals guessed via button press whether the value of a visually-presented card was high or low (4 seconds: presentation of a question mark). An expectancy cue was then presented for 2, 4 or 6 seconds with four types of cues/trial types described below. The outcome then appeared for 1 second (the number for 500ms and then the feedback arrow for 500ms), followed by a 0.5-1.5 second inter-trial interval (ITI). The four trial types were as follows: expectation of possible win, followed by win outcome (win trials: $1 reward) or no change (disappointment trials); expectation of possible loss, followed by loss (loss trials: 75 cent loss) or no change (relief trials); mixed win/loss trials, followed by win or loss; and neutral trials, followed by no change, with 12 trials for each of the 4 trial types per block. The paradigm was administered in two blocks of 8 minutes and including 48 trials per block. Subjects received $1 for each win and 75 cents deducted for each loss, and total possible earnings were $6. The order of trials was randomly determined across participants.

The following events were modelled within an SPM first level model, using the canonical hemodynamic response function (HRF): gambling stimulus, expectancy cue, outcome. Reward expectancy and outcome uncertainty were included as parametric modulators coupled to the expectancy cue, while reward prediction error was included as a parametric modulator at the outcome cue. Omission errors, where made, were also modelled.

**EFNback:** The EFNBACK task is an n-back paradigm including emotional distractors (Bertocci et al., 2023). The n-back task included two memory load conditions: a low-memory load (0-back-e.g., press the button to “M”) and high memory load (2-back-e.g., press the button whenever the letter is identical to the letter presented in two trials back (L-X-L)). Each memory load condition included one of four emotional face distractor conditions (fearful, happy, neutral, or no face distractor). Two blocks of 7min 4seconds were administers, including 24 blocks of 12 500ms long trials each. During a jittered inter-trial interval (mean duration = 3500ms), a fixation cross flanked with faces was displayed. Participants were instructed to respond as quickly as possible with their index finger to the target letter. Brief instructions are presented on the screen for 4000ms at the beginning of each block. Detailed instructions are provided during task practice prior to the scanning session. Incorrect trials were excluded from the analysis. For each of the two blocks, eight conditions were modelled within an SPM first level model using the canonical HRF: the two memory loads (0-, 2-back) across the four face conditions. Two further conditions – errors and instructions – were also modelled.

**Dynamic Faces:** A 12.5-minute emotional dynamic faces task was performed (Greenberg et al., 2017). Stimuli comprised faces from the NimStim set (Tottenham et al., 2009) that were morphed in 5% increments, from neutral (0% emotion) to 100% emotion for four emotions: happy, sad, angry, and fear. Morphed faces were collated into one-second movies progressing from 0% to 100% emotional display. In control trials, movies comprised a simple shape (dark oval) superimposed on a light-grey oval, with similar structural characteristics to the face stimuli, which subsequently morphed into a larger shape, approximating the movement of the morphed faces. An additional set of trials included neutral faces that morphed from one identity to another. There were three blocks for each of the four emotional conditions with 12 stimuli per block, and six control blocks with six stimuli per block. Emotional and control blocks were presented in a pseudo-randomized order so that no two blocks of any condition were presented sequentially. Participants were asked to use a button press, selecting one of three buttons, to indicate the color of a semi-transparent foreground color flash (orange, blue, or yellow) that appeared during the mid-200–650msec of the one-second presentation of the dynamically changing face. Each type of emotional face was modelled, as well as the neutral identity morphs and the shape conditions, within an SPM first level model using the canonical HRF. In addition, a second set of regressors was modelled using the HRF derivative.

**Resting:** Participants were asked to lie still and look at a cross hair on the screen while neuroimaging data were collected for six minutes.

*Data preprocessing*

Briefly, a volume-based normalization was performed using fMRIprep to normalize the structural and functional images into MNI space at a 2mm resolution. Slice timing correction and fieldmap distortion correction were not performed due to data formatting issues for some subjects. In addition, the fMRIprep CompCor method was not used for this study – see main text for details of nuisance correction. The following text represents automatically generated boilerplate text from the software. Results included in this manuscript come from preprocessing performed using fMRIPrep 20.2.6 (Esteban, Markiewicz, et al. (2018); Esteban, Blair, et al. (2018); RRID:SCR_016216), which is based on Nipype 1.7.0 (Gorgolewski et al. (2011); Gorgolewski et al. (2018); RRID:SCR_002502).

*Anatomical data preprocessing*

A total of 1 T1-weighted (T1w) images were found within the input BIDS dataset. The T1-weighted (T1w) image was corrected for intensity non-uniformity (INU) with N4BiasFieldCorrection (Tustison et al. 2010), distributed with ANTs 2.3.3 (Avants et al. 2008, RRID:SCR_004757), and used as T1w-reference throughout the workflow. The T1w-reference was then skull-stripped with a *Nipype* implementation of the antsBrainExtraction.sh workflow (from ANTs), using OASIS30ANTs as target template. Brain tissue segmentation of cerebrospinal fluid (CSF), white-matter (WM) and gray-matter (GM) was performed on the brain-extracted T1w using fast (FSL 5.0.9, RRID:SCR_002823, Zhang, Brady, and Smith 2001). Volume-based spatial normalization to two standard spaces (MNI152NLin6Asym, MNI152NLin2009cAsym) was performed through nonlinear registration with antsRegistration (ANTs 2.3.3), using brain-extracted versions of both T1w reference and the T1w template. The following templates were selected for spatial normalization: *FSL’s MNI ICBM 152 non-linear 6th Generation Asymmetric Average Brain Stereotaxic Registration Model* [Evans et al. (2012), RRID:SCR_002823; TemplateFlow ID: MNI152NLin6Asym], *ICBM 152 Nonlinear Asymmetrical template version 2009c* [Fonov et al. (2009), RRID:SCR_008796; TemplateFlow ID: MNI152NLin2009cAsym],

*Functional data preprocessing*

For each of the 6 BOLD runs found per subject (across all tasks and sessions), the following preprocessing was performed. First, a reference volume and its skull-stripped version were generated using a custom methodology of *fMRIPrep*. Susceptibility distortion correction (SDC) was omitted. The BOLD reference was then co-registered to the T1w reference using flirt (FSL 5.0.9, Jenkinson and Smith 2001) with the boundary-based registration (Greve and Fischl 2009) cost-function. Co-registration was configured with nine degrees of freedom to account for distortions remaining in the BOLD reference. Head-motion parameters with respect to the BOLD reference (transformation matrices, and six corresponding rotation and translation parameters) are estimated before any spatiotemporal filtering using mcflirt (FSL 5.0.9, Jenkinson et al. 2002). The BOLD time-series (including slice-timing correction when applied) were resampled onto their original, native space by applying the transforms to correct for head-motion. These resampled BOLD time-series will be referred to as *preprocessed BOLD in original space*, or just *preprocessed BOLD*. The BOLD time-series were resampled into standard space, generating a *preprocessed BOLD run in MNI152NLin6Asym space*. First, a reference volume and its skull-stripped version were generated using a custom methodology of *fMRIPrep*.

All resamplings can be performed with *a single interpolation step* by composing all the pertinent transformations (i.e. head-motion transform matrices, susceptibility distortion correction when available, and co-registrations to anatomical and output spaces). Gridded (volumetric) resamplings were performed using antsApplyTransforms (ANTs), configured with Lanczos interpolation to minimize the smoothing effects of other kernels (Lanczos 1964). Non-gridded (surface) resamplings were performed using mri_vol2surf (FreeSurfer).

Many internal operations of *fMRIPrep* use *Nilearn* 0.6.2 (Abraham et al. 2014, RRID:SCR_001362), mostly within the functional processing workflow. For more details of the pipeline, see [the section corresponding to workflows in *fMRIPrep*’s documentation](https://fmriprep.readthedocs.io/en/latest/workflows.html).

### Nipype References

Abraham, Alexandre, Fabian Pedregosa, Michael Eickenberg, Philippe Gervais, Andreas Mueller, Jean Kossaifi, Alexandre Gramfort, Bertrand Thirion, and Gael Varoquaux. 2014. “Machine Learning for Neuroimaging with Scikit-Learn.” *Frontiers in Neuroinformatics* 8. <https://doi.org/10.3389/fninf.2014.00014>.

Avants, B.B., C.L. Epstein, M. Grossman, and J.C. Gee. 2008. “Symmetric Diffeomorphic Image Registration with Cross-Correlation: Evaluating Automated Labeling of Elderly and Neurodegenerative Brain.” *Medical Image Analysis* 12 (1): 26–41. <https://doi.org/10.1016/j.media.2007.06.004>.

Behzadi, Yashar, Khaled Restom, Joy Liau, and Thomas T. Liu. 2007. “A Component Based Noise Correction Method (CompCor) for BOLD and Perfusion Based fMRI.” *NeuroImage* 37 (1): 90–101. <https://doi.org/10.1016/j.neuroimage.2007.04.042>.

Esteban, Oscar, Ross Blair, Christopher J. Markiewicz, Shoshana L. Berleant, Craig Moodie, Feilong Ma, Ayse Ilkay Isik, et al. 2018. “FMRIPrep.” *Software*. Zenodo. <https://doi.org/10.5281/zenodo.852659>.

Esteban, Oscar, Christopher Markiewicz, Ross W Blair, Craig Moodie, Ayse Ilkay Isik, Asier Erramuzpe Aliaga, James Kent, et al. 2018. “fMRIPrep: A Robust Preprocessing Pipeline for Functional MRI.” *Nature Methods*. <https://doi.org/10.1038/s41592-018-0235-4>.

Evans, AC, AL Janke, DL Collins, and S Baillet. 2012. “Brain Templates and Atlases.” *NeuroImage* 62 (2): 911–22. <https://doi.org/10.1016/j.neuroimage.2012.01.024>.

Fonov, VS, AC Evans, RC McKinstry, CR Almli, and DL Collins. 2009. “Unbiased Nonlinear Average Age-Appropriate Brain Templates from Birth to Adulthood.” *NeuroImage* 47, Supplement 1: S102. <https://doi.org/10.1016/S1053-8119(09)70884-5>.

Gorgolewski, K., C. D. Burns, C. Madison, D. Clark, Y. O. Halchenko, M. L. Waskom, and S. Ghosh. 2011. “Nipype: A Flexible, Lightweight and Extensible Neuroimaging Data Processing Framework in Python.” *Frontiers in Neuroinformatics* 5: 13. <https://doi.org/10.3389/fninf.2011.00013>.

Gorgolewski, Krzysztof J., Oscar Esteban, Christopher J. Markiewicz, Erik Ziegler, David Gage Ellis, Michael Philipp Notter, Dorota Jarecka, et al. 2018. “Nipype.” *Software*. Zenodo. <https://doi.org/10.5281/zenodo.596855>.

Greve, Douglas N, and Bruce Fischl. 2009. “Accurate and Robust Brain Image Alignment Using Boundary-Based Registration.” *NeuroImage* 48 (1): 63–72. <https://doi.org/10.1016/j.neuroimage.2009.06.060>.

Jenkinson, Mark, Peter Bannister, Michael Brady, and Stephen Smith. 2002. “Improved Optimization for the Robust and Accurate Linear Registration and Motion Correction of Brain Images.” *NeuroImage* 17 (2): 825–41. <https://doi.org/10.1006/nimg.2002.1132>.

Jenkinson, Mark, and Stephen Smith. 2001. “A Global Optimisation Method for Robust Affine Registration of Brain Images.” *Medical Image Analysis* 5 (2): 143–56. <https://doi.org/10.1016/S1361-8415(01)00036-6>.

Lanczos, C. 1964. “Evaluation of Noisy Data.” *Journal of the Society for Industrial and Applied Mathematics Series B Numerical Analysis* 1 (1): 76–85. <https://doi.org/10.1137/0701007>.

Power, Jonathan D., Anish Mitra, Timothy O. Laumann, Abraham Z. Snyder, Bradley L. Schlaggar, and Steven E. Petersen. 2014. “Methods to Detect, Characterize, and Remove Motion Artifact in Resting State fMRI.” *NeuroImage* 84 (Supplement C): 320–41. <https://doi.org/10.1016/j.neuroimage.2013.08.048>.

Reuter, Martin, Herminia Diana Rosas, and Bruce Fischl. 2010. “Highly Accurate Inverse Consistent Registration: A Robust Approach.” *NeuroImage* 53 (4): 1181–96. <https://doi.org/10.1016/j.neuroimage.2010.07.020>.

Satterthwaite, Theodore D., Mark A. Elliott, Raphael T. Gerraty, Kosha Ruparel, James Loughead, Monica E. Calkins, Simon B. Eickhoff, et al. 2013. “An improved framework for confound regression and filtering for control of motion artifact in the preprocessing of resting-state functional connectivity data.” *NeuroImage* 64 (1): 240–56. <https://doi.org/10.1016/j.neuroimage.2012.08.052>.

Tustison, N. J., B. B. Avants, P. A. Cook, Y. Zheng, A. Egan, P. A. Yushkevich, and J. C. Gee. 2010. “N4ITK: Improved N3 Bias Correction.” *IEEE Transactions on Medical Imaging* 29 (6): 1310–20. <https://doi.org/10.1109/TMI.2010.2046908>.

Zhang, Y., M. Brady, and S. Smith. 2001. “Segmentation of Brain MR Images Through a Hidden Markov Random Field Model and the Expectation-Maximization Algorithm.” *IEEE Transactions on Medical Imaging* 20 (1): 45–57. <https://doi.org/10.1109/42.906424>.

**Supplementary Discussion**

Our findings have implications from the point of view of reproducibility. First, the GS summary statistic associations with sex replicated quite precisely across measures: although our criterion for replication was a significant p value, we were able to recapitulate the original effect size estimate in the independent sample. Thus, our findings are consistent with recent machine learning studies suggesting effective classification of a participant’s sex from neuroimaging measures (Al Zoubi et al., 2022; Gallo et al., 2023; Ryali, Zhang, de Los Angeles, Supekar, & Menon, 2024; Weis et al., 2020), and may provide insight into what neural features contribute to this classification. One recent development in this regard has been to start to evaluate sex-specific models of brain/behavior associations (Dhamala, Jamison, Jaywant, & Kuceyeski, 2022; Dhamala et al., 2023; Dong et al., 2024), which has identified sex differences in the prediction of behavioral or clinical variables using FC measures. In general, it seems likely that these modelling considerations may be theoretically salient in the context of mental illnesses which show sex differences including major depression (Nolen-Hoeksema, 1987) and ADHD (Arnett, Pennington, Willcutt, DeFries, & Olson, 2015).

Second, there was striking variability in the reliability of given FC measures or regional variances. Regional variance was generally in the moderate/good range, while FC was generally poor (Cicchetti, 1994). However, there was considerable variation in FC reliability, and some FC measures reached the ‘good’ range. Notably, regions selected on the basis of their reliability showed good replication of the PCA-derived factors across samples. Ideally, for Drysdale-like findings (Drysdale et al., 2017) to be observed reliably across cohorts, factor replication of this sort would be necessary to support reliable PLS or CCA models. However, the null findings with the ICC selection method showed that this was not related to individual differences in our behavioral measures across subjects. Indeed, the reliability of an FC measure or regional variance, and its association with behavioral measures, were largely unrelated. This suggests that there are clear determinants of individual differences in neural activity which are unrelated to the behavioral measures we selected, and that modelling these effectively may be necessary to uncover reliable brain/behavior relationships. One such determinant appeared to be the region in question, with reproducible variation across FC measures/regions. Intriguingly, many of the more reliable regions / FC measures were situated in cortical areas which might subserve cognitive function, and this reliability might contribute to the relatively effective prediction of cognition from FC (Ooi et al., 2022). Whether our observation of regionally-consistent FC or variance is related to our choice of paradigms and our decision to regress out task-related activation might be evaluated in future work. In addition, the use of a multiband sequence might have had a negative impact on subcortical relative to cortical reliability (Srirangarajan, Mortazavi, Bortolini, Moll, & Knutson, 2021).

In general, our analytic approach was biased towards the identification of specific effects, including the regional and frequency specificity of FC, as well as more specific phenotypic dimensions. It might reasonably be argued that our correction method was too stringent, with only equivocal and/or uncorrected findings being observed. Ultimately, the present method would only work effectively if a subset of FC measures or region variances were strongly and reproducibly related to the constructs of interest, and be largely unaffected by GSR. A possible alternative scenario regarding the relation of affective symptoms or traits to FC is described by Cremers and colleagues (Cremers, Wager, & Yarkoni, 2017): namely, relatively weak and distributed effects, rather than strong, localized findings. Our study cannot confirm the latter i.e. presence of relationships of a particular FC measure with specific traits/symptoms related to mood disorders (e.g. anhedonia, behavioral inhibition). Thus, possible explanations of the Drysdale findings may involve distributed relationships. In addition, less specific symptoms of MDD may also be relevant including sequalae of illness severity e.g. cognitive dysfunction or somatic symptoms, perhaps relating to arousal including fatigue. Indeed, given that cognitive impairment is a comorbidity of major depression (Rock, Roiser, Riedel, & Blackwell, 2014), individual differences in cognition may contribute to the capacity of FC-based machine learning methods to categorize MDD and controls (but see Zhukovsky et al., 2022). Overall, the present findings may help to narrow down the range of likely brain/behavior relationships relevant for disorders with an affective component.

**Other References**

Al Zoubi, O., Misaki, M., Tsuchiyagaito, A., Zotev, V., White, E., Paulus, M., & Bodurka, J. (2022). Machine Learning Evidence for Sex Differences Consistently Influences Resting-State Functional Magnetic Resonance Imaging Fluctuations Across Multiple Independently Acquired Data Sets. *Brain Connect, 12*(4), 348-361. doi:10.1089/brain.2020.0878

Arnett, A. B., Pennington, B. F., Willcutt, E. G., DeFries, J. C., & Olson, R. K. (2015). Sex differences in ADHD symptom severity. *J Child Psychol Psychiatry, 56*(6), 632-639. doi:10.1111/jcpp.12337

Bertocci, M. A., Afriyie-Agyemang, Y., Rozovsky, R., Iyengar, S., Stiffler, R., Aslam, H. A., . . . Phillips, M. L. (2023). Altered patterns of central executive, default mode and salience network activity and connectivity are associated with current and future depression risk in two independent young adult samples. *Mol Psychiatry, 28*(3), 1046-1056. doi:10.1038/s41380-022-01899-8

Chase, H. W., Fournier, J. C., Bertocci, M. A., Greenberg, T., Aslam, H., Stiffler, R., . . . Phillips, M. L. (2017). A pathway linking reward circuitry, impulsive sensation-seeking and risky decision-making in young adults: identifying neural markers for new interventions. *Transl Psychiatry, 7*(4), e1096. doi:10.1038/tp.2017.60

Cicchetti, D. V. (1994). Guidelines, criteria, and rules of thumb for evaluating normed and standardized assessment instruments in psychology. *Psychological Assessment, 6*(4), 284-290.

Cremers, H. R., Wager, T. D., & Yarkoni, T. (2017). The relation between statistical power and inference in fMRI. *PLoS One, 12*(11), e0184923. doi:10.1371/journal.pone.0184923

Dhamala, E., Jamison, K. W., Jaywant, A., & Kuceyeski, A. (2022). Shared functional connections within and between cortical networks predict cognitive abilities in adult males and females. *Hum Brain Mapp, 43*(3), 1087-1102. doi:10.1002/hbm.25709

Dhamala, E., Rong Ooi, L. Q., Chen, J., Ricard, J. A., Berkeley, E., Chopra, S., . . . Holmes, A. J. (2023). Brain-Based Predictions of Psychiatric Illness-Linked Behaviors Across the Sexes. *Biol Psychiatry, 94*(6), 479-491. doi:10.1016/j.biopsych.2023.03.025

Dong, D., Pizzagalli, D. A., Bolton, T. A. W., Ironside, M., Zhang, X., Li, C., . . . Belleau, E. L. (2024). Sex-specific resting state brain network dynamics in patients with major depressive disorder. *Neuropsychopharmacology, 49*(5), 806-813. doi:10.1038/s41386-024-01799-1

Drysdale, A. T., Grosenick, L., Downar, J., Dunlop, K., Mansouri, F., Meng, Y., . . . Liston, C. (2017). Resting-state connectivity biomarkers define neurophysiological subtypes of depression. *Nat Med, 23*(1), 28-38. doi:10.1038/nm.4246

Gallo, S., El-Gazzar, A., Zhutovsky, P., Thomas, R. M., Javaheripour, N., Li, M., . . . van Wingen, G. (2023). Functional connectivity signatures of major depressive disorder: machine learning analysis of two multicenter neuroimaging studies. *Mol Psychiatry*. doi:10.1038/s41380-023-01977-5

Greenberg, T., Bertocci, M. A., Chase, H. W., Stiffler, R., Aslam, H. A., Graur, S., . . . Phillips, M. L. (2017). Mediation by anxiety of the relationship between amygdala activity during emotion processing and poor quality of life in young adults. *Transl Psychiatry, 7*(7), e1178. doi:10.1038/tp.2017.127

Nolen-Hoeksema, S. (1987). Sex differences in unipolar depression: evidence and theory. *Psychol Bull, 101*(2), 259-282.

Ooi, L. Q. R., Chen, J., Shaoshi, Z., Kong, R., Tam, A., Li, J., . . . Yeo, B. T. T. (2022). Comparison of individualized behavioral predictions across anatomical, diffusion and functional connectivity MRI. *Neuroimage*, 119636. doi:10.1016/j.neuroimage.2022.119636

Rock, P. L., Roiser, J. P., Riedel, W. J., & Blackwell, A. D. (2014). Cognitive impairment in depression: a systematic review and meta-analysis. *Psychol Med, 44*(10), 2029-2040. doi:10.1017/S0033291713002535

Ryali, S., Zhang, Y., de Los Angeles, C., Supekar, K., & Menon, V. (2024). Deep learning models reveal replicable, generalizable, and behaviorally relevant sex differences in human functional brain organization. *Proc Natl Acad Sci U S A, 121*(9), e2310012121. doi:10.1073/pnas.2310012121

Srirangarajan, T., Mortazavi, L., Bortolini, T., Moll, J., & Knutson, B. (2021). Multi-band FMRI compromises detection of mesolimbic reward responses. *Neuroimage, 244*, 118617. doi:10.1016/j.neuroimage.2021.118617

Tottenham, N., Tanaka, J. W., Leon, A. C., McCarry, T., Nurse, M., Hare, T. A., . . . Nelson, C. (2009). The NimStim set of facial expressions: judgments from untrained research participants. *Psychiatry Res, 168*(3), 242-249. doi:10.1016/j.psychres.2008.05.006

Weis, S., Patil, K. R., Hoffstaedter, F., Nostro, A., Yeo, B. T. T., & Eickhoff, S. B. (2020). Sex Classification by Resting State Brain Connectivity. *Cereb Cortex, 30*(2), 824-835. doi:10.1093/cercor/bhz129

Zhukovsky, P., Wainberg, M., Milic, M., Tripathy, S. J., Mulsant, B. H., Felsky, D., & Voineskos, A. N. (2022). Multiscale neural signatures of major depressive, anxiety, and stress-related disorders. *Proc Natl Acad Sci U S A, 119*(23), e2204433119. doi:10.1073/pnas.2204433119
